# Development and Testing of Force Field Parameters for Phenylalanine and Tyrosine Derivatives

**DOI:** 10.3389/fmolb.2020.608931

**Published:** 2020-12-15

**Authors:** Xiaowen Wang, Wenjin Li

**Affiliations:** ^1^Institute for Advanced Study, Shenzhen University, Shenzhen, China; ^2^College of Physics and Optoelectronic Engineering, Shenzhen University, Shenzhen, China

**Keywords:** unnatural amino acids, charge parameters, Amber ff14SB, relative energy, molecular dynamics, MM/PBSA

## Abstract

Theoretical analyses are valuable for the exploration of the effects of unnatural amino acids on enzyme functions; however, many necessary parameters for unnatural amino acids remain lacking. In this study, we developed and tested force field parameters compatible with Amber ff14SB for 18 phenylalanine and tyrosine derivatives. The charge parameters were derived from ab initio calculations using the RESP fitting approach and then adjusted to reproduce the benchmark relative energies (at the MP2/TZ level) of the α- and β-backbones for each unnatural amino acid dipeptide. The structures optimized under the proposed force field parameters for the 18 unnatural amino acid dipeptides in both the α- and β-backbone forms were in good agreement with their QM structures, as the average RMSD was as small as 0.1 Å. The force field parameters were then tested in their application to seven proteins containing unnatural amino acids. The RMSDs of the simulated configurations of these unnatural amino acids were approximately 1.0 Å compared with those of the crystal structures. The vital interactions between proteins and unnatural amino acids in five protein–ligand complexes were also predicted using MM/PBSA analysis, and they were largely consistent with experimental observations. This work will provide theoretical aid for drug design involving unnatural amino acids.

## Introduction

As is well known, 20 natural amino acids are the main building blocks of proteins, the macromolecules that perform a broad spectrum of functions within organisms (Qin et al., 2015). Unnatural amino acids (UAAs) also called noncanonical amino acids are analogs or metabolic intermediates of the 20 natural amino acids with only minor structural differences—often just a chemical functional group—which is beneficial for analyzing their effects on enzyme functions (Zhao et al., 2020). Since UAAs are of high chemical diversity, possess strong site specificity, and introduce little disturbance to the protein structure, it is widely applied in protein engineering, virus vaccine development, and medical therapeutics (Minnihan et al., 2011; Si et al., 2016; Young and Schultz, 2018). For instant, biological catalysis and reaction mechanism of tyrosine in aminoacyl-tRNA synthetases (aaRS) were investigated through the incorporation of UAA fluorotyrosine, whose pKa was tuned by changing the number and the site of fluoro-substitution (Minnihan et al., 2011). Si and co-workers employed the UAA N^ε^-2-azidoethyloxycarbonyl-l-lysine to produce replication-incompetent viral vaccines by introducing premature termination codon into the genome of influenza A virus, and these viral vaccines prevented further damage inside conventional cells via immune response (Si et al., 2016). In addition, UAAs are utilized in the bio-orthogonal reactivity. For example, UAA-incorporated proteins (such as antibodies, growth factors, and cytokines) specifically interacted with diverse moieties to form bispecific antibodies, antibody-drug conjugates, and pegylated proteins, which provided effective treatments for various clinical testing (Young and Schultz, 2018).

The incorporation of UAAs into canonical proteins expanded significantly the genetic code library (Xiao et al., 2015). Natural UAAs occur commonly in plants, microorganisms, and animals, while those in human organisms must be chemically synthesized (Zou et al., 2018). Typically, an orthogonal amber suppressor aaRS/tRNA pair has been utilized to guide the incorporation of UAAs in response to a unique nonsense codon (Santoro et al., 2002; Liu and Schultz, 2010). Experimentally, many studies have reported the incorporation of UAAs into the designated sites of target proteins by means of popular residue-specific and site-directed mutagenesis approaches (Sakamoto et al., 2002; Fleissner et al., 2009; Xiao et al., 2015; Yuet et al., 2015). For example, Yuet et al. described a method for residue-specific labeling that enabled the use of the UAA *p*-azido-l-phenylalanine (AzF) to tag and analyze protein metabolism in specific cells based on the phenylalanyl-tRNA synthetase (Yuet et al., 2015). Schultz et al. utilized site-directed mutagenesis to mutate Val216 of TEM-1 β-lactamase into *p*-acrylamido-phenylalanine (AcrF), which enhanced the catalytic activity of the enzyme (Xiao et al., 2015). Although the two complementary methods involved in residue-specific and site-directed mutagenesis are widely used to incorporate UAAs into proteins, they often contend with certain technical difficulties.

To compensate for experimental obstacles, theoretical computational methods validated by experimental data offer a novel way to screen potential analogs for natural amino acids. A number of computational methods to study proteins containing UAAs have been successively reported by other groups in recent years (Renfrew et al., 2012; Petrov et al., 2013; Khoury et al., 2014a,b). For example, Renfrew et al. constructed a rotamer library containing 114 UAAs to study the interface of calpain and calpastatin, which was evaluated using a scoring function based on the Rosetta program (Leaverfay et al., 2011; Renfrew et al., 2012). New GROMOS54a7 force field parameters were developed by the Zagrovic group for processing post-translationally modified amino acids by means of molecular dynamics (MD) simulations executed by the GROMACS package (Petrov et al., 2013). In addition, a tool called “Forcefield_NCAA” created by the Floudas lab is now available for generating UAA parameters related to a library of 147 noncanonical amino acids compatible with the Amber ff03 parameters (Khoury et al., 2014a,b).

**Figure 1:**
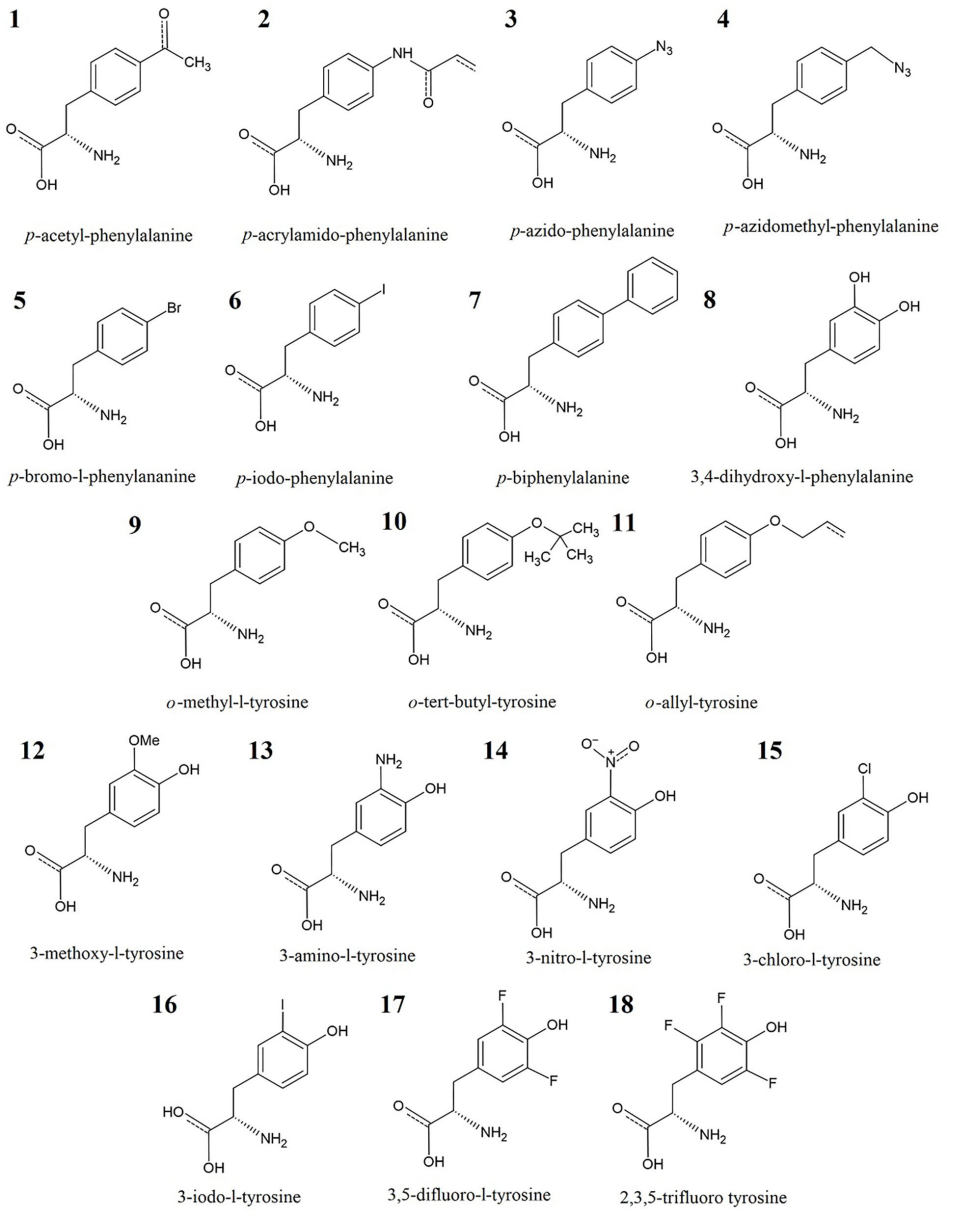
Training set of 18 UAAs: analogs of phenylalanine (No. 1–8) and tyrosine (No. 9–18).

The aim of our work was to develop and test force field parameters for phenylalanine and tyrosine derivatives, most of which are not included in the reported literature. The structures of the involved UAAs in this study are displayed in Figure 1. The newly developed parameters were then applied to mutant proteins or protein–ligand interactions involving UAAs, as listed in Supplementary Table 1, by MD simulations and molecular mechanics–Poisson Boltzmann solvent accessible surface area (MM/PBSA) calculations. Based on comparison with experimental data as the benchmark, the simulation results indicate that the new force field parameters can predict protein structures with incorporated UAAs well and generally describe the exact interplay that occurs in the binding pockets of proteins with UAAs as substrates.

## Simulation Strategies

**Figure 2:**
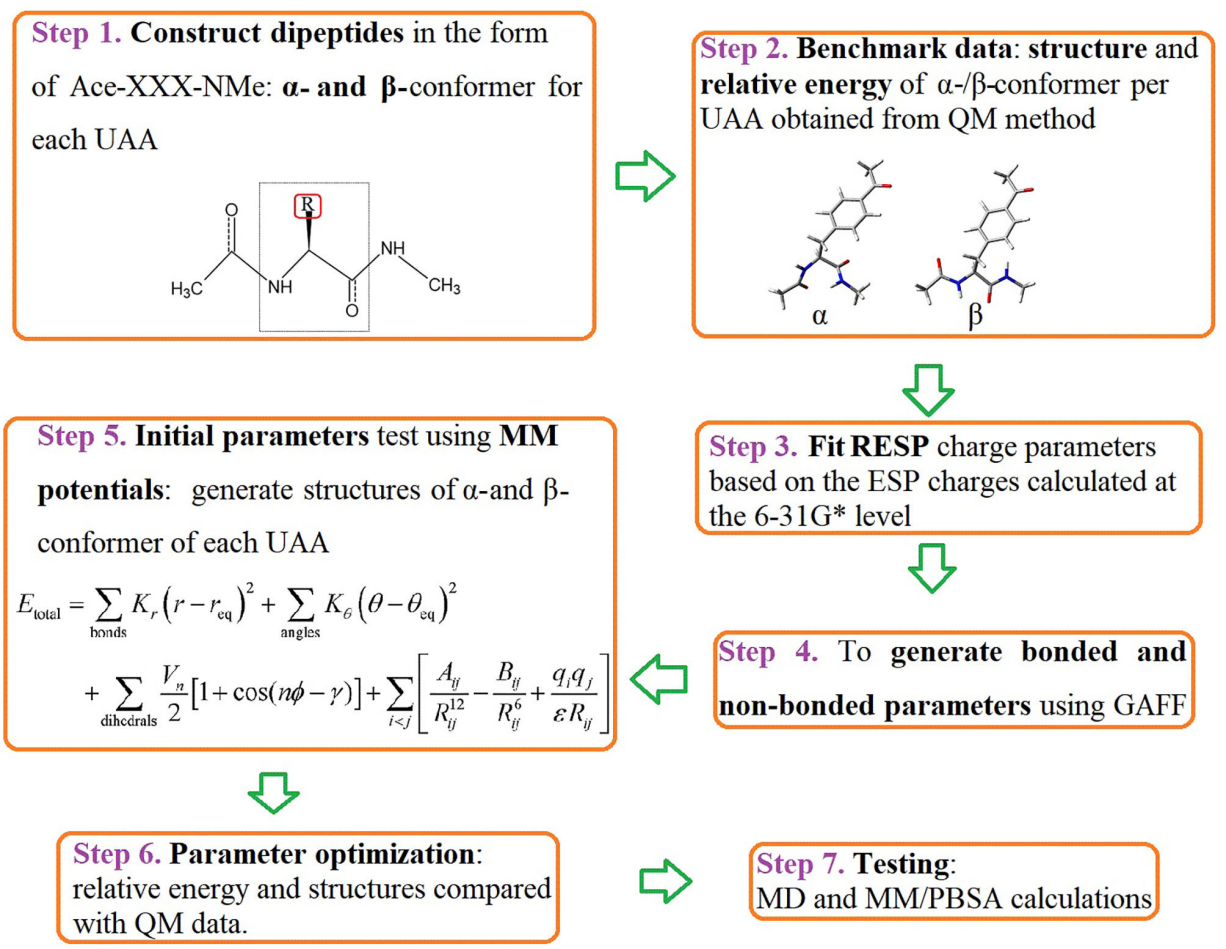
Workflow for parametrization of the 18 UAAs. The diagram describes the current protocol for parameter derivation and testing for the selected phenylalanine and tyrosine derivatives. Key words for each step are indicated in bold.

We first constructed dipeptides of the α- and β-conformer of each UAA in the form of Ace-XXX-NMe using GaussView 6 (Dennington et al., 2016) (Step 1 in Figure 2). Here, XXX represents the analogs of phenylalanine and tyrosine shown in Figure 1. It is a popular way to employ α- and β-backbones of amino acids to fit parameters in current classical force fields, such as AMBER, CHARMM, and OPLS (Hornak et al., 2006; Best et al., 2012; Robertson et al., 2015), as these backbones dominate in the sterically allowed structural regions of the Ramachandran plot (Ramachandran et al., 1963). For the constructed dipeptides, structural optimization was performed at the B3LYP/6-31G^*^ level, and single-point energy calculations were executed at the MP2/cc-pVTZ level using the Gaussian 16 program (Step 2 in Figure 2) (Frisch et al., 2016). Based on the optimized structures obtained at the B3LYP/6-31G^*^ level, the electrostatic potential (ESP) charges at the HF/6-31G^*^ level were further evaluated; this is a popular method to produce ESP charges because of the accurate reproduction of free energies of solvation and liquid enthalpies (Cornell et al., 1993; Wang et al., 2000). In Step 3, restrained electrostatic potential (RESP) charges were generated based on the ESP-fit charge model (Cornell et al., 1995). The general Amber force field (GAFF) is a useful molecular mechanics and is designed to be suitable for organic molecules, especially drug-like small molecules (Wang et al., 2004). In the following stage, we thus produced bonded and non-bonded parameters using GAFF based on the Antechamber tool (Case et al., 2020). The newly generated parameters can be transferred into the GMX format using the ACPYPE.py script for subsequent MD simulations in the GROMACS software package (Sousa da Silva and Vranken, 2012). In Step 5, the initial parameters of the structures of the α- and β-conformers of each UAA were tested. Accordingly, we optimized the charge parameters of the 18 analogs by estimating the relative energies of each UAA pair compared with the benchmark of quantum mechanics (QM) data at the MP2/cc-pVTZ level. In the final step, MD simulations and MM/PBSA calculations on the proteins or protein–ligand complexes involving UAAs were further performed to test the new parameters determined in this work. The complete workflow of parametrization is shown in Figure 2, and the detailed methodology for producing the parameters is described below.

### QM Calculations

The 18 UAAs shown in Figure 1 are analogs derived from the amino acids of phenylalanine (F) and tyrosine (Y). Based on the 18 UAAs, we constructed dipeptides of two backbone conformers for each UAA blocked with *N*-methyl and acetyl groups in the form of Ace-XXX-NMe in GaussView 6 (Dennington et al., 2016). The two backbone conformers were designed in the forms of an α-helix (ϕ = −60°, ψ = −40°) and β-strand (ϕ = −180°, ψ = 180°). Structural optimizations were performed at the B3LYP/6-31G^*^ level (Mondal et al., 2007), followed by single-point energy calculations at the MP2/cc-pVTZ level (Harder et al., 2016). For comparison, an additional method for the structure and energy calculations was performed at the M06-2X level (Robertson et al., 2015). The pseudopotential for iodine-containing systems was assigned as the SDD basis set in this work (Yurieva et al., 2008). The missing van der Waals (vdW) radius for iodine atoms was chosen as the Pauling radius (2.15 Å) (Pauling, 1939). The QM calculations were performed using the Gaussian 16 program (Frisch et al., 2016).

### Energy Model

The total pair potential energy used in this work is written as a sum of terms as follows:

(1) $$\begin{array}{r} E_{total}=E_{bond}+E_{angle}+E_{dihedral}+E_{es}+E_{vdW} \end{array}$$

Our goal is to develop UAA charge parameters that are compatible with the Amber ff14SB parameter set for the 20 natural amino acids. The energy function (Equation 1) from the Amber force field is thus employed here (Maier et al., 2015). Generally, the vdW radius and epsilon parameters are derived from experimental data (Weiner et al., 1984). The charge parameters were adjusted using the protocol described below.

### Parameter Optimization

The partial charges were fitted using RESP charges obtained at the HF/6-31G^*^ level (Cornell et al., 1993, 1995; Wang et al., 2000). The initial bonding and vdW parameters were generated from GAFF using the Antechamber module in AmberTools20 (Case et al., 2020). The charge sets of the Ace and NMe groups are identical to the Amber ff14sb force fields. Together, we used bonded and non-bonded parameters to calculate the structures and relative energies of the α- and β-conformers of the 18 UAAs. By comparing the QM structures and relative energies, we adjusted the charge parameters of the UAA backbones and side chains until good accordance was achieved with the QM data in terms of the root-mean-square (RMS) deviation, as shown in Equation 2.

(2) $$\begin{array}{r} \text{RMS}=\sqrt{\frac{\overset{N}{\underset{i=1}{\sum }}{\left(RE_{QM}^{}\left(i\right)-RE_{our}^{}\left(i\right)\right)}^{2}}{N}} \end{array}$$

where *RE*_QM_(*i*) and *RE*_our_(*i*) are the relative energies calculated by QM and the new parameters developed in our work for the *i*th training set, respectively, and *N* is the total number of training sets. We minimized the RMS values to obtain the charge parameters.

### MD Simulations

MD simulations were performed using the 2019 version of the GROMACS program (Abraham et al., 2015). We chose the Amber ff14SB force field for proteins composed of natural amino acids (Maier et al., 2015). For the UAA components, the new parameters developed in this work were used. We placed the initial systems in the center of a cubic box 10 Å from the box edge. The box was then filled with a water solvent using the TIP3P water model (Jorgensen et al., 1983). The water molecules were randomly replaced by Na^+^ and Cl^−^ ions to a 0.1 M concentration. For each model, energy minimization with a maximum of 5,000 steps was carried out without any restraints. After optimization, two short 200 ps MD simulations in the NVT and NPT ensembles were successively performed with the heavy-atom position restraint at a force constant of 500 kcal/(mol·Å^2^). The position restraints were gradually released via four steps of 100 ps NPT simulations with force constants of 250, 100, 50, and 10 kcal/(mol·Å^2^) for the heavy atoms. Finally, 20 ns production MD simulations were performed in the NPT ensemble. The time step was set to 2 fs, and the temperature and pressure were kept constant at 300 K and 1 bar, respectively. In the production runs, the velocity-rescaling thermostat was applied for temperature coupling (Berendsen et al., 1984; Bussi et al., 2007), while the Parrinello–Rahman approach was applied for constant pressure control (Parrinello and Rahman, 1981; Nosé and Klein, 1983). The SHAKE algorithm was used to constrain covalent bonds involving hydrogen atoms (Andersen, 1983; Miyamoto and Kollman, 1992). The particle mesh Ewald method was applied to the calculation of long-range electrostatic interactions (Darden et al., 1993). The cutoff values for vdW and electrostatic forces were set to 12 Å, and the simulation structures were saved every 100 ps to obtain the trajectories for analysis.

### MM/PBSA Estimation

In general, the binding free energy for protein–ligand interactions can be expressed as

(3) $$\begin{array}{r} \Delta G_{\text{bind}}=\Delta E_{\text{vdW}}+\Delta E_{\text{ele}}+\Delta G_{\text{solv}}-T\Delta S \end{array}$$

where Δ*E*_vdW_ and Δ*E*_ele_ are the non-bonded terms of the system total energy (Δ*E*_MM_) due to vdW and electrostatic interactions, respectively. The bonded terms of Δ*E*_MM_ were assumed to be zero in the single-trajectory setup used in this procedure because of its simplicity and accuracy similar to those of a multi-trajectory setup (Genheden and Ryde, 2015; Wang et al., 2018). Δ*G*_solv_ is the solvation free energy required to move the solute from a vacuum (dielectric constant of 1) into the solvent (dielectric constant of 80). It can be further decomposed into polar (Δ*G*_pb/solv_) and nonpolar (Δ*G*_np/solv_) contributions to solvation. *T* and Δ*S* are the absolute temperature and entropy, respectively. However, the entropy term was ignored in this study because of the significant time consumption, uncertainty of the contributions to the total free energy, and small improvement by comparison with the experimental results (Yang et al., 2011; Kumari et al., 2014).

Furthermore, the binding free energy decomposition of each residue was analyzed to understand the key residue impact at the activation region of the protein–inhibitor interaction. Hence, the free energy of each residue ($\Delta G_{\text{res}}^{\text{bind}}$) can be divided into three terms:

(4) $$\begin{array}{r} \Delta G_{\text{res}}^{\text{bind}}=\Delta E_{\text{res}}^{\text{MM}}+\Delta G_{\text{res}}^{\text{pb/solv}}+\Delta G_{\text{res}}^{\text{np/solv}} \end{array}$$

where $\Delta E_{\text{res}}^{\text{MM}}$ is the sum of the electrostatic and vdW interactions per residue in a vacuum, and $\Delta G_{\text{res}}^{\text{pb/solv}}$ and $\Delta G_{\text{res}}^{\text{np/solv}}$ are the polar and nonpolar parts of the per-residue solvation free energy, respectively.

In this work, the successive 20 ns trajectories produced were used to perform MM/PBSA calculations on the free energies using the g_mmpbsa tool (Kumari et al., 2014). Here, the system coordinates were saved for every 1 ns used for MM/PBSA analysis such that 20 snapshots for each trajectory were considered to calculate the binding free energies of the protein–inhibitor interactions. The Poisson–Boltzmann (PB) equation was applied to calculate Δ*G*_pb/solv_ (Honig and Nicholls, 1995). The temperature and grid spacing were set to 300 K and 0.5 Å, respectively, and the concentration of charged ions was 0.1 M with radii of 0.95 and 1.81 Å for Na^+^ and Cl^−^, respectively. The solvent accessible surface area (SASA) model was employed to estimate the nonpolar contributions (Δ*G*_np/solv_) from the function γSASA + *b* (Sitkoff et al., 1994). The radius value for SASA was 1.4 Å, and the constants γ and *b* were set to default values of 0.00542 kcal/(mol·Å^2^) and 0.92 kcal/mol, respectively.

## Results and Discussion

### Initial Parameters Applied to α-/β-Conformer Optimization

**Figure 3:**
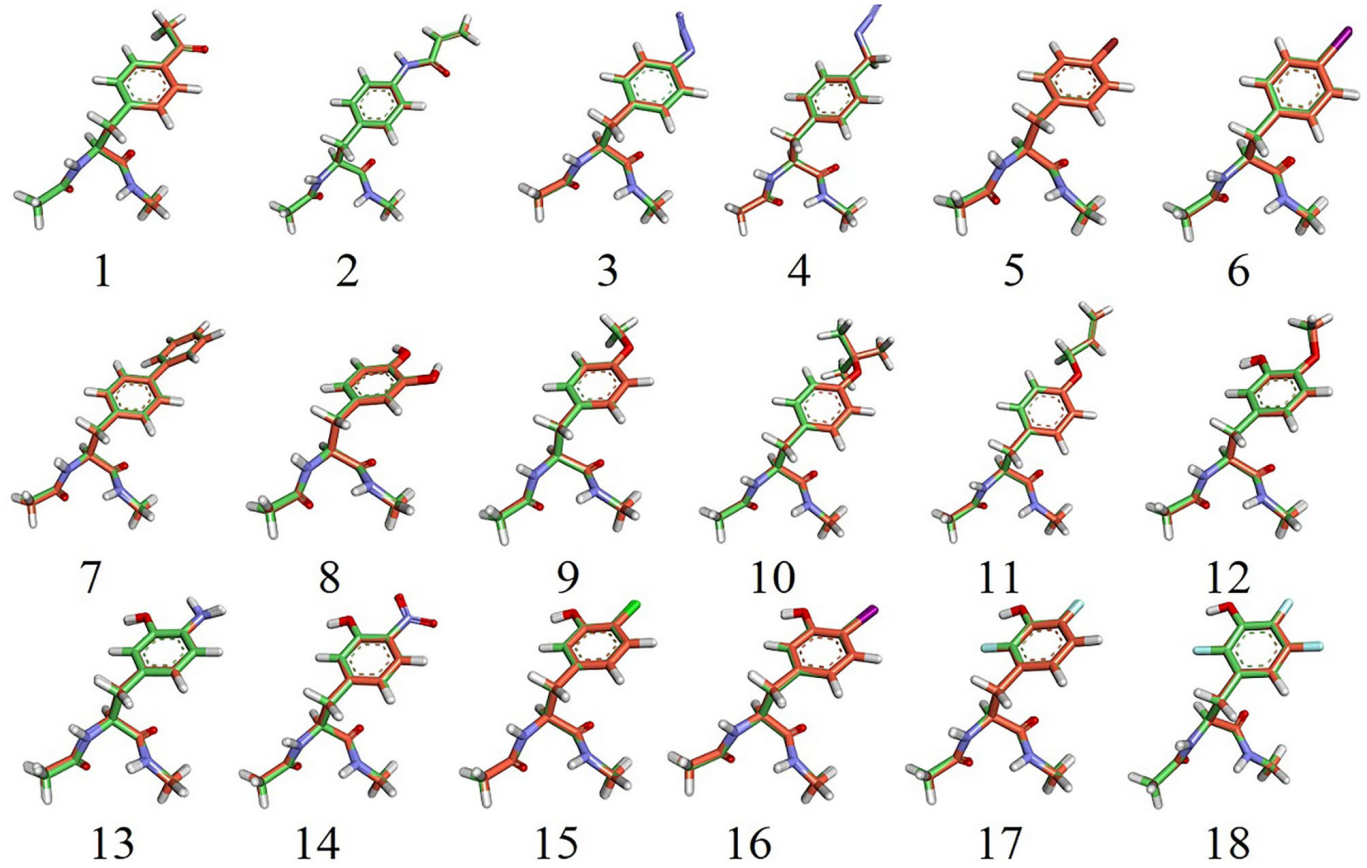
Overlap of 18 α-backbone conformations after energy minimization of the QM (B3LYP/6-31G*) structures. N, O, and H atoms are shown in blue, red, and white, respectively. C atoms from the simulation and QM structures are shown in green and orange, respectively. F(17 and 18), Cl(15), Br(5), and I(6, 16) atoms are shown in cyan, green, red, and magenta, respectively.

After the initial parameters (hereafter referred to as cycle-1 parameters) involved in the bonded and non-bonded terms were generated, we performed structural optimizations for the α-and β-conformers of each UAA. For comparison with the B3LYP/6-31G^*^ structures, we depict the optimized structures of the 18 UAA dipeptides in the α-state from the initial parameters in Figure 3; the minimized structures for the β-state are shown in Supplementary Figure 1. As shown, the two backbone conformations in the α- and β-states of the training set are in good agreement with the QM structures. The initial parameters also performed well for the side-chain structures. Additionally, the determined heavy-atom and all-atom RMS displacements (RMSDs) for the 18 training sets from Table 1 are nearly <0.1 Å (refer also to the RMSD distributions in Supplementary Figure 2). Among the systems, system 13 has the greatest RMSDs of 0.083–0.116 Å. Meanwhile, Supplementary Figure 2 shows that the all-atom RMSDs are comparable to the heavy-atom RMSDs but fluctuate to slightly higher values. Overall, the initial parameters yield good results for the 18 training sets, especially the bonded connections, but further improvements to the energies are necessary.

**Table 1:** Initial parameter test for the 18 training sets evaluated by heavy-atom and all-atom RMSDs.

**Training set**	**Heavy-atom RMSD** **(Å)**		**All-atom RMSD** **(Å)**	
	**α-Backbone**	**β-Backbone**	**α-Backbone**	**β-Backbone**
1	0.041	0.052	0.045	0.059
2	0.072	0.055	0.083	0.060
3	0.054	0.052	0.051	0.053
4	0.046	0.039	0.043	0.046
5	0.068	0.052	0.067	0.058
6	0.080	0.042	0.079	0.048
7	0.084	0.041	0.082	0.053
8	0.084	0.045	0.082	0.057
9	0.046	0.046	0.048	0.053
10	0.044	0.042	0.048	0.048
11	0.054	0.045	0.060	0.051
12	0.064	0.048	0.068	0.059
13	0.096	0.083	0.116	0.094
14	0.056	0.047	0.058	0.055
15	0.073	0.055	0.074	0.063
16	0.072	0.058	0.075	0.065
17	0.067	0.040	0.069	0.046
18	0.046	0.039	0.047	0.045

### Testing of Optimized Parameters

Displayed in Table 2 are the relative energies for the 18 training sets. We selected the relative energies evaluated at the MP2/cc-pVTZ//B3LYP/6-31G^*^ level of theory as a benchmark (Mondal et al., 2007; Harder et al., 2016). For comparison, one density functional theory (DFT) method with a small basis set at the M06-2X/6-311++G^**^//M06-2X/6-31+G^*^ level was used in this work (Robertson et al., 2015). For the parameter optimization process, four cycles were performed. First, we fixed the charges of the Ace and NMe groups in the 18 UAA dipeptides to remain the same as the corresponding Amber ff14sb force field parameter sets and made minor adjustments to the backbone RESP charges. As shown in Table 2, the relative energies from the cycle-1 parameters show a correlation of 0.8212 compared with the MP2 energies, with a larger RMS deviation of 4.86 kcal/mol. In the next two cycles, we chose to treat the backbones and side chains as α-helical RESP charges and averaged RESP charges in the α- and β-states, respectively. In the third cycle adjustment, the RMS decreased to 2.33 kcal/mol with a 0.8072 correlation. At this point, we noted that the relative energies of most systems were comparable to the benchmark data except for those of systems 4, 9–12, 17, and 18. Therefore, the parameters for these systems were further optimized. In the final procedure, we chose the β-conformational charges from the first cycle as the determined parameters for systems 4 and 18. For systems 9–12 and 17, different proportions between the α- and β-conformational RESP charges were ultimately treated (see the footnotes in Table 2). For the remaining systems, we employed the averaged charges in the α- and β-states. Eventually, we observed a strong correlation between our work and the QM data, with *R*^2^ = 0.9407 (Supplementary Figure 3). Therefore, the parameters from the fourth cycle were employed in subsequent calculations. Although the partial charges were obtained by fitting to the RESP of independent conformations for each UAA, the partial charges of the atoms in their common structures are quite close to each other (see Supplementary Material). Note that these UAAs are phenylalanine and tyrosine derivatives and share a common structure. The observation of such small differences indicates that the obtained RESPs for these UAAs are reliable and the charge parameters are well converged.

**Table 2:** Relative energies (kcal/mol) for the α- and β-conformers of the 18 UAA dipeptides obtained from QM calculations and our work.

**Training set**	**MP2^**a**^**	**M06-2X^**b**^**	**Our work**			
			**Cycle 1**	**Cycle 2**	**Cycle 3**	**Cycle 4^**c**^**
1	6.50	7.39	11.07	2.61	6.72	6.72
2	6.93	7.72	10.78	3.34	7.12	7.12
3	6.35	7.13	11.14	1.96	6.70	6.70
4	6.05	7.30	6.63	0.01	3.52	6.63
5	6.45	7.28	10.11	2.01	6.31	6.31
6	6.53	7.38	10.56	2.44	6.55	6.55
7	6.76	7.58	10.36	2.77	6.76	6.76 (5.74)
8	6.77	7.54	10.06	3.18	7.06	7.06
9	6.86	7.62	12.78	5.56	9.57	7.00 (8.52)
10	6.89	9.74	12.40	5.54	9.06	6.77
11	6.79	7.54	12.32	4.33	8.22	7.10 (8.05)
12	8.02	8.90	15.12	6.34	11.08	7.93
13	8.26	8.99	13.78	4.05	8.94	8.94 (4.40)
14	7.12	8.08	14.38	2.44	7.70	7.70
15	7.44	8.37	12.76	1.80	7.58	7.58
16	7.53	8.42	12.88	2.69	7.86	7.86
17	5.07	5.54	6.70	−6.75	0.47	4.96
18	4.80	5.34	4.45	−7.52	−1.53	4.45
^d^RMS MP2	–	1.08	4.86	5.68	2.33	0.33
^e^*R*^2^ (MP2)		0.7831	0.8212	0.6922	0.8072	0.9407

a MP2/cc-pVTZ//B3LYP/6-31G^*^.

b M06-2X/6-311++G^**^//M06-2X/6-31+G^*^.

c Values in parentheses were obtained using charge parameters taken from the literature (Khoury et al., 2014b). The different proportions of charge parameters in the final cycle are β/6 + α^*^5/6 for system 9, β/5 + α^*^4/5 for system 10, β/3 + α^*^2/3 for system 11, β/6 + α^*^5/6 for system 12, β^*^7/8 + α/8 for system 17, β for systems 4 and 18, and β/2 + α/2 for the other systems.

d *All units for RMS deviations are kcal/mol*.

e *Correlation between MP2 and the other methods involved in M06-2X and our work*.

*The relative energy (RE) is defined as E_α_ – E_β_*.

In addition, we noted that the β-backbone conformation of each UAA is more stable than the α-backbone as predicted by all employed methods. Here, our work shows a more favorable RMS deviation of 0.33 kcal/mol compared with M06-2X with an RMS deviation of 1.08 kcal/mol. Additionally, existing charge parameters from a reference were also tested on reported systems 7, 9, 11, and 13 (Khoury et al., 2014b). The relative energies of these four systems are 5.74, 8.52, 8.05, and 4.40 kcal/mol obtained from the reported parameters, which are comparable to those from the MP2 data of 6.76, 6.86, 6.79, and 8.26 kcal/mol, respectively, but produce absolute errors of approximately 1.5 kcal/mol or higher (Supplementary Table 2). Compared with the MP2 energies, as also shown in Supplementary Table 2, the RMS deviations obtained from the reference and our work were 2.25 and 0.38 kcal/mol, respectively, for these four systems. Therefore, the energetic performance of the new parameters determined in our work results in more satisfactory predictions. In addition, the structural optimizations from the cycle-4 parameters were again tested on the 18 dipeptides in the α- and β-states. Comparisons of heavy-atom RMS distributions between cycles 1 and 4 are provided in Supplementary Figure 4, which clearly shows that the new parameters produce smaller heavy-atom RMS deviations than the initial parameters. Overall, the new parameters show a good performance in terms of structural optimization and relative energy calculations for the 18 UAA models based on comparison with QM results, indicating that the new parameters determined in this work are appropriate for performing further tests via MD simulations.

### Testing

#### MD Simulations of Proteins Containing UAAs

Seven isolated protein systems containing UAAs were selected to identify the new parameters as the testing set. At present, crystals composed of noncanonical amino acids have rarely been recorded in the PDB. We attempted to search for the protein structures covering UAAs related to phenylalanine and tyrosine, which are T4 lysozyme (PDB ID: 3HWL) (Fleissner et al., 2009), CaM-peptide (PDB ID: 6HCS) (Creon et al., 2018), modified threonyl-tRNA synthetase (PDB ID: 4S0I) (Pearson et al., 2015), sphingosine-1-phosphate lyase (PDB ID: 3MBB) (Bourquin et al., 2010), birch pollen allergen Bet v 1.0101 (PDB ID: 4B9R) (Ackaert et al., 2014), ketosteroid isomerase (PDB ID: 5D82) (Wu et al., 2015), and acetyltransferase (PDB ID: 2Z10) (Sakamoto et al., 2009). Each protein mainly contains one UAA, dominated by secondary structures of α-helices, β-sheets, and γ-turns made of natural amino acids. Among them, the UAAs ACF131, AZF108, NIY150, CHY16, and IOY111 incorporated in the T4 lysozyme, CaM-peptide, Bet v 1.0101, ketosteroid isomerase, and acetyltransferase, respectively, are mainly located at the α-helices. BFA11 and NIY5, 66, and 83 of the threonyl-tRNA synthetase and Bet v 1.0101 are distributed in the β-sheet regions, and AMY249 of sphingosine-1-phosphate lyase is located at the γ-turn. The remaining five systems involving UAAs recorded in the PDB are in regard to the protein–ligand interactions (Supplementary Table 1) and will be discussed in the next section, “MM/PBSA analysis of protein–UAA interactions.”

**Figure 4:**
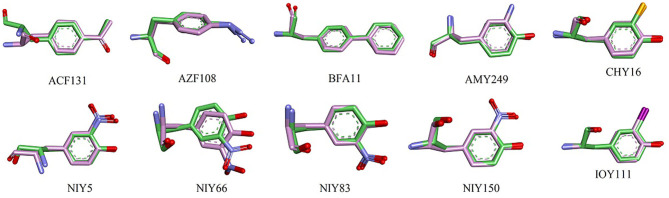
Structural alignment between crystal and MD stable structures of single UAAs in isolated protein systems. C atoms from the crystal and MD stable structures are shown in green and pink, respectively. All N and O atoms are blue and red, and Cl and I atoms from CHY16 and IOY111 are orange and magenta, respectively.

Here, we show each UAA fragment from the final MD structure compared with the crystal structure in Figure 4, and Table 3 displays the averaged heavy-atom RMSD values of the UAAs and corresponding proteins in isolated systems. As shown in Figure 4, the backbone and side chains of the UAAs in the isolated proteins are generally well overlapped with the experimental structures, although there are slight structural derivations in the fragments of the ACF131 backbone and NIY66 side chain. Table 3 also shows that the averaged RMSDs for ACF131 and NIY66 are the largest at 1.31 ± 0.11 and 0.95 ± 0.20 Å, respectively, corresponding to moderate RMSDs of 1.94 ± 0.24 and 2.43 ± 0.29 Å for their whole proteins. Simultaneously, we inspected the UAA motions by referring to one equilibrium structure during MD simulations, as listed in Table 3 (column 3). Almost all the RMSDs of the UAAs are under 0.5 Å, with only NIY5 showing a larger RMSD of 0.66 ± 0.30 Å. In addition, we plotted the RMSD distributions for each UAA in the isolated protein systems compared with the crystal structures over time in Supplementary Figure 5. As shown, each trajectory reaches a balance after 20 ns MD simulations. However, the RMSD of NIY5 decreased by <0.5 Å between 5 and 10 ns. After 10 ns, all the UAAs reached equilibrium with RMSDs under 1.5 Å.

**Table 3:** Averaged heavy-atom RMSDs (Å) with standard errors of the mean for the UAAs and corresponding proteins in isolated systems.

**UAA**	**RMSD** **(Å)**			**PDB ID**
	**UAA fit to crystal UAA**	**UAA fit to MD UAA**	**Protein fit to backbone**	
ACF131	1.31 ± 0.11	0.39 ± 0.39	1.94 ± 0.24	3HWL
AZF108	0.75 ± 0.11	0.28 ± 0.13	3.44 ± 0.31	6HCS
BFA11	0.22 ± 0.06	0.23 ± 0.07	1.75 ± 0.15	4S0I
AMY249	0.42 ± 0.12	0.23 ± 0.09	4.36 ± 0.48	3MBB
NIY5	0.72 ± 0.29	0.66 ± 0.30	2.43 ± 0.29	4B9R
NIY66	0.95 ± 0.20	0.40 ± 0.25	2.43 ± 0.29	4B9R
NIY83	0.30 ± 0.09	0.23 ± 0.08	2.43 ± 0.29	4B9R
NIY150	0.28 ± 0.07	0.31 ± 0.09	2.43 ± 0.29	4B9R
CHY16	0.26 ± 0.01	0.24 ± 0.10	2.80 ± 0.25	5D82
IOY111	0.15 ± 0.04	0.14 ± 0.04	2.02 ± 0.20	2Z10

**Figure 5:**
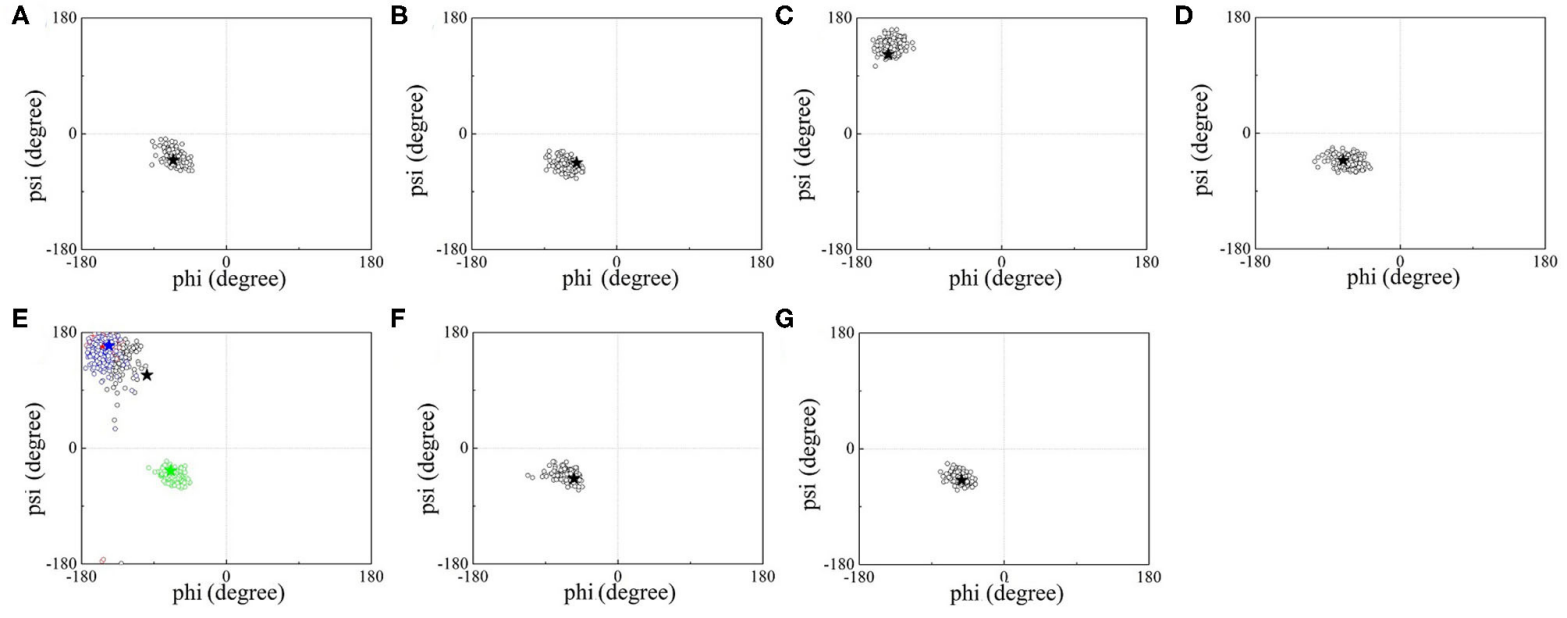
ϕ/ψ backbone torsional statics for **(A)** ACF, **(B)** AZF, **(C)** BFA, **(D)** AMY, **(E)** NIY, **(F)** CHY, and **(G)** IOY during the MD simulations. Black hollow circles describe the torsional distributions of ϕ/ψ over time, and black stars indicate the crystal data for the corresponding UAAs. Four NIY structures are contained in **(E)**, where black, red, blue, and green circles correspond to the backbone torsional distributions of NIY5, 66, 83, and 150, respectively, and the four colored stars indicate the corresponding crystal structures.

Additionally, the backbone conformations of seven UAAs in their isolated proteins obtained from the new parameters during the MD simulations were further investigated (Figure 5). As shown in Figure 5E, only NIY5 of birch pollen allergen Bet v 1.0101 (PDB ID: 4B9R) inclines toward the more stretched β-sheet backbone conformation during the MD simulation (black symbols). Compared to the crystal structures, the calculated backbone torsions of the remaining UAAs are generally well consistent. The backbones of ACF131, AZF108, AMY249, NIY150, CHY16, and IOY111 are in the form of α-helices, while those of BFA and NIY5, 66, and 83 are formed by β-strands.

#### MM/PBSA Analysis of Protein–UAA Interactions

Aside from the isolated proteins containing UAAs found in the PDB search, the UAAs were resolved as a ligand role in protein–UAA interactions (Turner et al., 2006; Moor et al., 2011; Takimoto et al., 2011; Li et al., 2013). To evaluate the quality of the new parameters determined in this work, five systems of protein–UAA interactions were studied by MM/PBSA analysis. The complexes are *p*-bromo-l-phenylalanine (BRF) bound to aaRS (PDB ID: 2AG6) (Turner et al., 2006); tRNA^Phe^ with 3,4-dihydroxy-l-phenylalanine (DHF) (PDB ID: 3TEG) (Moor et al., 2011); evolved PylRS charged with *o*-methyl-l-tyrosine (OMY) (PDB ID: 3QTC) (Takimoto et al., 2011); tyrosine-tRNA ligase mutant complexed with 3-methyl-tyrosine (MEY) (PDB ID: 4HPW); and 3,5-difluoro-l-tyrosine (DFY) incorporated into tyrosine phosphorylation (PDB ID: 4HJX) (Li et al., 2013). In addition, we added H and OH groups to the -NH and -C=O termini, respectively, to achieve neutral UAA ligands. We made minor modifications to the charge parameters of the terminal H and OH groups; the modified terminal charges for the H and OH groups of the five ligands BRF, DHF, OMY, MEY, and DFY are listed in Supplementary Table 3. These charge parameters should be more appropriate for UAAs when they are treated as ligands.

**Figure 6:**
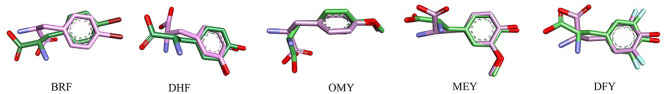
Structural alignment between crystal and MD stable structures of single UAAs in protein–ligand interactions. F and Br atoms from DFY and BRF are light blue and dark red, respectively. The colors of other atoms are the same as in Figure 4.

Compared with the UAAs incorporated into isolated proteins, the UAAs involved as substrates in protein–ligand interactions seem to shift more obviously, particularly the backbone structures (Figure 6). This may be due to the flexible UAA structures acting as ligands to bind with the proteins. The average RMSD values were also calculated to be larger, around 1.3 Å, as listed in Table 4. After choosing one equilibrium MD structure as the reference, the averaged RMSDs for all UAA ligands decreased to below 1.0 Å, suggesting good stability in the simulation process. Supplementary Figure 6 plots the RMSD distributions as a function of time for the five ligands BRF, DHF, OMY, MEY, and DFY during the MD simulation starting from the experimental structure set. As shown, the DHF, OMY, MEY, and DFY ligands were well-balanced after 3 ns, whereas BRF reached another stable state after 10 ns. The RMSD values of all the UAA ligands are in the vicinity of 1.5 Å, showing stable movements over the initial structures. The final whole structures also overlap well with the crystal structures, as depicted in Supplementary Figures 7H–L, indicating that our new parameters can reproduce the experimental structures of these protein–UAA interactions.

**Table 4:** Averaged heavy-atom RMSDs (Å) with standard errors of the mean for UAAs and corresponding proteins in protein–ligand complexes.

**UAA**	**RMSD**			**PDB ID**
	**UAA fit to crystal UAA**	**UAA fit to MD UAA**	**Protein fit to backbone**	
BRF	0.96 ± 0.37	0.83 ± 0.51	2.23 ± 0.15	2AG6
DHF	1.33 ± 0.16	0.81 ± 0.49	2.42 ± 0.15	3TEG
OMY	1.33 ± 0.27	0.87 ± 0.46	2.38 ± 0.20	3QTC
MEY	1.31 ± 0.38	0.61 ± 0.44	2.44 ± 0.22	4HPW
DFY	1.11 ± 0.27	0.47 ± 0.31	3.29 ± 0.30	4HJX

Further, we used the MD structures obtained from the new parameters to calculate the five protein–UAA complexes. Table 5 shows the binding free energies of aaRS–BRF (PDB ID: 2AG6), tRNA^Phe^-DHF (PDB ID: 3TEG), PylRS–OMY (PDB ID: 3QTC), tRNA^Tyr^-MEY (PDB ID: 4HPW), and tyrosine phosphorylation (F2YRS)–DFY (PDB ID: 4HJX) based on MM/PBSA analysis. The binding free energy of 3TEG is the highest at −21.3 kcal/mol, while the weakest binding affinity of −6.4 kcal/mol corresponds to 2AG6. As mentioned above, the RMSD of BRF reaches another local equilibrium within a period of 12–20 ns. To check the convergence of the MM/PBSA calculation, the results were usually estimated from different time intervals (Spiliotopoulos et al., 2012). As shown in Supplementary Table 4, the binding free energy of 2AG6 estimated using the 12–20 ns trajectory is −7.1 kcal/mol, which is largely consistent with the one obtained using entire trajectory and the one in an early stage. We also predicted the binding free energies of tRNA^Tyr^-MEY (4HPW) and tyrosine phosphorylation–DFY (4HJX) as −17.3 and −13.6 kcal/mol, respectively. The energy decomposition analysis also indicates that vdW and electrostatic interactions are the dominate factors contributing to the total binding free energy. The polar energies contribute positively to the solvation. Overall, the binding free energies for the five systems were well stabilized by the MM contributions.

**Table 5:** Binding free energies (kcal/mol) with standard deviation^a^ for the systems 2AG6, 3TEG, 3QTC, 4HPW, and 4HJX obtained from MM/PBSA calculations and various energy components.

**Component**	**2AG6**	**3TEG**	**3QTC**	**4HPW**	**4HJX**
Δ*E*_vdW_	−25.2(0.4)	−24.3(0.5)	−20.6(1.4)	−25.4(0.9)	−26.4(0.6)
Δ*E*_ele_	−11.8(1.5)	−35.6(1.7)	−12.7(1.5)	−20.2(1.9)	−18.8(1.5)
Δ*G*_pb/solv_	33.6(1.5)	41.4(1.4)	20.0(1.1)	31.1(1.6)	34.7(1.5)
Δ*G*_np/solv_	−3.0(0.0)	−2.7(0.0)	−2.4(0.1)	−2.9(0.0)	−3.1(0.0)
Δ*G*_pb_	21.8(0.0)	5.8(0.2)	7.3(0.2)	10.9(0.2)	15.9(0.0)
Δ*G*_np_	−28.2(0.2)	−27.0(0.3)	−23.0(0.8)	−28.3(0.6)	−29.5(0.3)
Δ*E*_MM_	−37.0(0.7)	−59.9(0.8)	−33.3(0.0)	−45.6(0.6)	−45.2(0.5)
Δ*G*_solv_	30.6(0.9)	38.7(0.8)	17.6(0.6)	28.2(0.9)	31.6(0.9)
Δ*G*_bind_	−6.4(0.8)	−21.2(1.1)	−15.7(1.0)	−17.4(1.1)	−13.6(0.8)

a *The standard deviations are calculated by the equation $\text{SD}=\sqrt{\overset{N}{\underset{i=1}{\sum }}{\left(x_{i}-\bar{x_{i}}\right)}^{2}/N}$*.

*Here, ΔG_pb_ = ΔG_pb/solv_ + ΔE_ele_; ΔG_np_ = ΔG_np/solv_ + ΔE_vdW_; ΔE_MM_ = ΔE_vdW_ + ΔE_ele_; ΔG_solv_ = ΔG_pb/solv_ + ΔG_np/solv_*.

In addition, the binding affinities from experimental data for two of the complexes are available. One is the reported Michaelis constant *K*_M_ for the system tRNA^Phe^-DHF (3TEG) of 380 ± 40 μM (Moor et al., 2011), which determines the performance of the catalytic reaction and positively correlates with the dissociation constant *K*_d_ (Johnson and Goody, 2011). The other is for OMY as one of the compstatin variants reported as *K*_d_ = 118 nM and ΔG = −9.5 ± 1.2 kcal/mol (Magotti et al., 2009). The binding free energy of PylRS and OMY interaction is predicted to be −15.7 ± 0.7 kcal/mol by MM/PBSA, which is in satisfying agreement with the experimental one. No experimental data of the binding affinity is available to date for the other three protein-UAA systems (PDB IDs: 2AG6, 4HPW, and 4HJX). Nevertheless, MM/PBSA analysis has been demonstrated to be an effective approach to estimate qualitatively the relative binding free energy of protein-ligand interaction (Homeyer and Gohlke, 2012; Kumari et al., 2014; Genheden and Ryde, 2015; Wang et al., 2019).

#### Per-Residue Energy Decomposition Analysis of Protein–UAA Interactions

**Figure 7:**
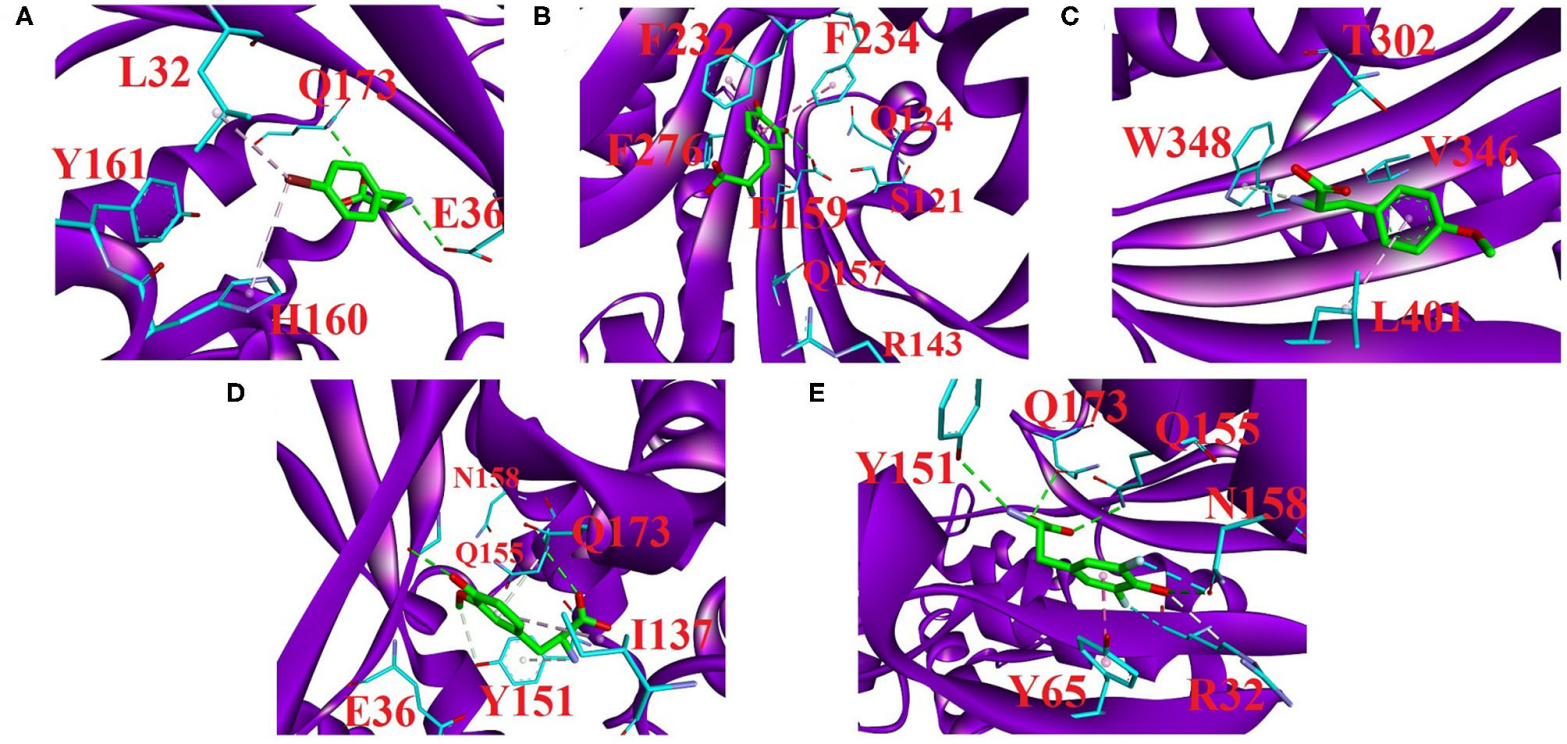
Major residue contacts with the substrates **(A)** BRF, **(B)** DHF, **(C)** OMY, **(D)** MEY, and **(E)** DFY in the active sites obtained by our predictions. The interaction analysis was completed using Discovery Studio 4.5 (BIOvIA, 2015). All C atoms in the substrates are shown as green sticks. The major interaction residues in the proteins (purple cartoon) are displayed as sticks with cyan C atoms. Red letters label the major residue names. Green dashed lines represent hydrogen bonding, and pink dashed lines represent the π-interaction involved in ligand recognition. The light pink in **(A)** and cyan in **(E)** dashed lines represent the halogen bonding occurring in the active regions.

The structural interaction modes between UAAs and proteins have been established by experimental reports (Turner et al., 2006; Moor et al., 2011; Takimoto et al., 2011; Li et al., 2013). We show the interaction details of the UAAs BRF, DHF, OMY, MEY, and DFY as substrates bound to the respective proteins in Figure 7. The per-residue binding free energies of the major contacts involved in the interactions are provided in Table 6. The interactions of the UAAs as substrates are discussed in the following sections.

**Table 6:** Energy decomposition analysis of 2AG6, 3TEG, 3QTC, 4HPW, and 4HJX for major residues.

**PDB ID**	**#Residue**	**Energy component (kcal/mol)**				**Standard deviation (kcal/mol)**			
		**MM**	**Polar**	**Non polar**	**Total**	**MM**	**Polar**	**Non polar**	**Total**
2AG6	**L32**	−1.26	1.15	−0.34	−0.45	0.38	0.31	0.03	0.48
	E36	−6.29	5.77	−0.33	−0.78	2.98	2.70	0.05	0.99
	**H160**	−2.24	4.85	−0.21	2.39	0.32	0.83	0.04	0.68
	**Y161**	−0.84	1.47	−0.17	0.47	0.12	0.20	0.02	0.19
	Q173	−4.31	10.22	−0.44	5.46	0.73	1.43	0.05	0.82
3TEG	**S121**	0.10	−0.40	−0.07	−0.36	0.21	0.38	0.02	0.25
	**Q124**	1.96	−0.39	−0.02	1.53	0.38	0.58	0.01	0.30
	**R143**	−3.66	7.75	−0.08	3.91	1.74	1.60	0.03	0.69
	**Q157**	−8.86	10.66	−0.58	1.18	1.12	0.85	0.04	0.75
	**E159**	−55.39	39.20	−0.38	−16.57	1.94	2.80	0.02	1.69
	**F232**	−4.07	2.03	−0.47	−2.53	0.60	0.38	0.03	0.35
	**F234**	−4.48	3.39	−0.55	−1.66	0.35	0.24	0.04	0.23
	F276	−6.78	3.94	−0.09	−2.91	0.40	0.40	0.02	0.45
3QTC	**T302**	−7.32	5.55	−0.39	−2.11	1.31	0.94	0.06	0.47
	**V346**	−2.03	0.26	−0.08	−1.84	0.34	0.09	0.02	0.38
	**W348**	−4.53	1.68	−0.15	−2.99	1.16	0.44	0.04	0.75
	**L401**	−4.10	0.68	−0.50	−3.91	0.77	0.19	0.07	0.67
4HPW	**E36**	−14.75	9.85	−0.52	−5.34	1.79	1.69	0.07	1.21
	I137	−4.56	0.75	−0.62	−4.43	0.37	0.35	0.04	0.25
	**Y151**	−13.06	7.57	−0.68	−6.17	0.95	0.78	0.06	1.09
	**Q155**	−9.69	8.13	−0.32	−1.90	1.19	0.96	0.05	0.45
	**N158**	−0.17	0.49	−0.02	0.31	0.21	0.49	0.02	0.34
	**Q173**	−17.58	16.48	−0.81	−1.97	1.55	0.87	0.06	1.02
4HJX	**R32**	−7.32	8.48	−0.27	0.82	0.71	1.19	0.03	0.98
	Y65	−7.78	4.41	−0.58	−3.91	0.68	0.47	0.05	0.56
	Y151	−6.95	6.62	−0.45	−0.76	0.65	0.62	0.05	0.50
	Q155	−23.04	21.54	−1.07	−2.59	1.59	1.62	0.06	0.67
	**N158**	−7.46	5.45	−0.28	−2.30	1.41	0.68	0.02	0.96
	Q173	−12.13	12.83	−0.50	0.20	1.49	1.17	0.03	0.71

*Bold letters represent residue contacts from experimental reports*.

##### aaRS and BRF interactions

In the 2AG6 system, our parameters predicted several direct connections of C–halogen-bonding interactions, which are consistent with the experimental results (Figure 7A). For example, the bromine of BRF forms a C-Br · · · π interaction with WT H160, which has been extensively reported in the crystal structures of protein–small molecules (Saraogi et al., 2003; Turner et al., 2006). One crystal structure report showed that the mutant L32 is a key mutant residue providing binding room for the bromine without vdW contributions (Turner et al., 2006). Here, the small contribution is −0.45 kcal/mol of free energy as predicted by our new parameters (see Table 6). In addition, we did not observe obvious contact between WT Y161 and BRF, and the predicted binding free energy was 0.47 kcal/mol, with weak contributions from MM, polar, and nonpolar interactions of −0.84, 1.47, and −0.17 kcal/mol, respectively. This is consistent with the experimental finding that the O atom of Y161 is too far (4.6 Å) to form H-bonded contact with the Br-atom of BRF in the active loop (Turner et al., 2006). In addition, two potential H-bonded contacts that have not been anticipated experimentally are predicted here. In particular, WT E36 and WT Q173 of 2AG6 use side chains to combine with the amide group of BRF in the form of H-bonds. As shown in Table 6, Q173 produces stronger polar interactions than the MM component, leading to a positive contribution of 5.46 kcal/mol. Strong electrostatic and vdW interactions of −6.29 and −4.31 kcal/mol were calculated for both E36 and Q173, respectively, which provide important conditions for H-bond formation (Li et al., 2014; Hao and Wang, 2015).

##### tRNA^Phe^ and DHF interactions

We provide the structural basis of the reported 3TEG (tRNA^Phe^ binding with DHF) in Figure 7B. F232 and F234 located at the FPF loop maintain major contacts with the phenyl ring of the ligand DHF (Moor et al., 2011). This was also observed from our predictions between F232/F234 and DHF in the form of π · · · π interactions. Simultaneously, F276 has a novel predicted role involved in DHF binding through an amide · · · π interaction (see Supplementary Figure 8A). These π-interaction modes formed by F232, F234, and F276 are similar to the reported “edge-to-face” contact (Fishman et al., 2001), an interaction network formed by three phenylalanine in tRNA^Phe^ binding with the phenyl moiety of the DHF ligand. The π · · · π and amide · · · π interactions mainly originate from vdW contributions (Gao et al., 2017). As shown in Table 6, the total vdW and electrostatic contributions of F232, F234, and F276 are all more negative than −4.0 kcal/mol. Furthermore, the binding free energy of E159 is a remarkable −16.57 kcal/mol, with surprisingly large non-bonded and polar contributions of −55.39 and 39.20 kcal/mol, respectively. As shown in Figure 7B, one hydrogen bonding connection occurs through the side-chain O atom of E159 with the negative charge binding to the OH group of DHF. Additionally, H-bonded connections have been reported between S121, Q124, R143, and Q157 in the protein and DHF shown in Supplementary Figure 8B (Moor et al., 2011). However, we failed to observe these hydrogen bonding contacts. Per-residue energy decomposition analysis further indicates that only R143 and Q157 provide dispensable non-bonded interactions of −3.66 and −8.86 kcal/mol, respectively. The contributions of S121 and Q124 are almost too weak for binding.

##### PylRS and OMY interactions

The four residue mutations in PylRS are A302T, N346V, C348W, and V401L, which play a vital role in the OMY selectivity (Takimoto et al., 2011). We also predicted these four important residue contacts with OMY based on the new parameters and per-residue binding free energy analysis. Figure 7C shows the interaction modes between OMY and the four residues T302, V346, W348, and LV401, and the binding free energy of each residue (PDB ID: 3QTC) is listed in Table 6. As shown, W348 uses a side-chain 5-membered ring as a π-donor to form hydrogen bonds with the N-atom in the amide group of OMY. This results in one quadrupole–dipole interaction formed by the indole plane of W348 being vertical to the O-methyl moiety of OMY (Takimoto et al., 2011). The binding free energy of W348 is −2.99 kcal/mol, providing strong vdW and electrostatic interactions of −4.53 kcal/mol. In the activation region, alkyl · · · π interactions occur by the methylene group of L401 binding with OMY, with the highest binding affinity contribution of −3.91 kcal/mol. Even though no direct connection forms between T302 and OMY, a moderate impact with a −2.11 kcal/mol binding free energy was evaluated, which also provides strong electrostatic and vdW interactions of −7.32 kcal/mol. In addition, the binding contribution of V346 is mainly derived from electrostatic and vdW contributions at −2.03 kcal/mol, but we did not observe hydrogen bonding between them. This is in agreement with the experimental observation that the H-bonds formed by WT N346 and OMY are abolished after the N346V mutation in PylRS (Takimoto et al., 2011).

##### tRNA^Tyr^ and MEY interactions

The structural basis for MEY recognition to tRNA^Tyr^ has not been reported to date, but the binding modes of the tRNA^Tyr^-MEY interaction can be analyzed and determined using the Mol^*^ tool provided in the PDB (Sehnal et al., 2018). Accordingly, E36, Y151, Q155, N158, and Q173 are the main residue contacts with MEY. Table 6 shows that the binding free energies of these residues provide negative contributions of −1.90 to −6.17 kcal/mol, except for N158 with 0.31 kcal/mol. Figure 7D displays the hydrogen bonding and alkyl · · · π interaction network between Y151, Q155, Q173, and MEY. Even though E36 does not form hydrogen bonding with MEY, a −5.34 kcal/mol strong affinity is derived from vdW and electrostatic attractions of −14.75 kcal/mol. Furthermore, I137 shows a new potential contact with MEY via an alkyl · · · π interaction with a −4.34 kcal/mol binding free energy.

##### F2YRS and DFY interactions

F2YRS shares approximately identical sequences with tRNA^Tyr^ except for the asparagine and cysteine at positions 108 and 109, respectively, corresponding to F108 and G109 in tRNA^Tyr^. The complex of F2YRS–DFY was obtained after Y32R, L65Y, H70G, F108N, Q109C, D158N, and L162S mutations by an experimental technique (Li et al., 2013). We assumed DFY to be in a neutral state due to the reported pKa value close to 7.0 (Seyedsayamdost et al., 2006). Figure 7E shows the six key residues binding to the DFY substrate. R32 and N158 form halogen bonding with the two different fluorine atoms of DFY; meanwhile, hydrogen bonding of R32 and N158 occurs with the OH group of DFY. This is consistent with experimental findings (Li et al., 2013). Experiments have also shown that there are strong dipolar interactions between the fluorine atoms and amide/guanidine groups. Notable polar contributions of 8.48 and 5.45 kcal/mol are estimated by our predictions for R32 and N158, respectively. In addition, Y65 forms π · · · π stacking interactions with the phenyl group of DFY, and Y151, Q155, and Q173 form hydrogen bonds with the amide and carbonyl groups of DFY. Among them, Q155 provides the largest MM contribution of −23.04 kcal/mol with 21.54 kcal/mol of polar energy. Y65 and Q155 with −3.91 and −2.59 kcal/mol free energies, respectively, contribute moderately to the observed binding.

## Conclusion

This work presents the charge parameters of 18 UAAs related to phenylalanine and tyrosine that are compatible with the use of the Amber ff14SB force field included in the GROMACS package. The newly derived charge parameters initially fitted by the RESP protocol were tested on structural optimizations and relative energies of the 18 UAAs in α-/β-backbone conformations, with an RMS deviation of 0.33 kcal/mol compared with the QM dataset, whereas the M06-2X method produces an RMS deviation of 1.08 kcal/mol. After the parameters were determined, the energy function was further applied to MD simulations of the UAA-mutated proteins and protein–UAA complexes. The motifs containing UAAs and their respective backbone torsions generally overlapped well with the initial coordinates, with an average RMSD of approximately 1.5 Å. The MM/PBSA approach showed that the binding free energy of tRNA^Phe^-DHF is higher than that of PylRS–OMY, which is consistent with experimental data. Comparisons with crystal residue contacts and satisfactory treatments for the interaction modes between proteins and UAAs by substrate binding are presented from the analysis of the per-residue energy decomposition.

Nevertheless, the development of force field is too far from only the development of charge parameters. To increase the transferability and compatibility to the standard Amber force field, the atoms in the common structure of these UAAs should be optimized to be of identical partial charges by applying restraints/constraints in the fitting to RESP in a future study. The bonded parameters, especially the torsional terms related to the gas-phase QM conformational potential energy scan, require further adjustment. The current testing concentrated on conformational and energetic investigations is also limited, and thus more extensive studies focusing on the dynamic and thermodynamic properties of polypeptides and proteins should be explored.

## Data Availability Statement

The original contributions generated for the study are included in the article/Supplementary Materials, further inquiries can be directed to the corresponding author/s.

## Author Contributions

WL proposed the core idea of the study. XW designed the project, performed the research, analyzed the data and drafted the manuscript. Both authors critically reviewed the manuscript.

## Conflict of Interest

The authors declare that the research was conducted in the absence of any commercial or financial relationships that could be construed as a potential conflict of interest.
