# Supplementary material for: Development and Testing of Force Field Parameters for Phenylalanine and Tyrosine Derivatives
Source: Front Mol Biosci. 2020 Dec 15;7:608931. doi: 10.3389/fmolb.2020.608931 (PMC7770134; doi:10.3389/fmolb.2020.608931)
Supplement: Supplementary file 1 [file Data_Sheet_1.PDF]

Supplementary Material

Development and Testing of Force Field Parameters for Phenylalanine and Tyrosine Derivatives

Xiaowen Wang[1,2] and Wenjin Li[1,*]

[1] Institute for Advanced Study, Shenzhen University, Shenzhen 518060, China

[2] College of Physics and Optoelectronic Engineering, Shenzhen University, Shenzhen 518060, China

[*]Correspondence:

Wenjin Li, Room 341, Administration Building, Institute for Advanced Study, Shenzhen University, Shenzhen 518060, China; E−mail: liwenjin@szu.edu.cn; Tel: +86−755−26942336

Contents:

Supplementary Figures

Supplementary Tables

Modified Charge Parameters for 18 UAAs (in GROMACS format)

Supplementary Figures

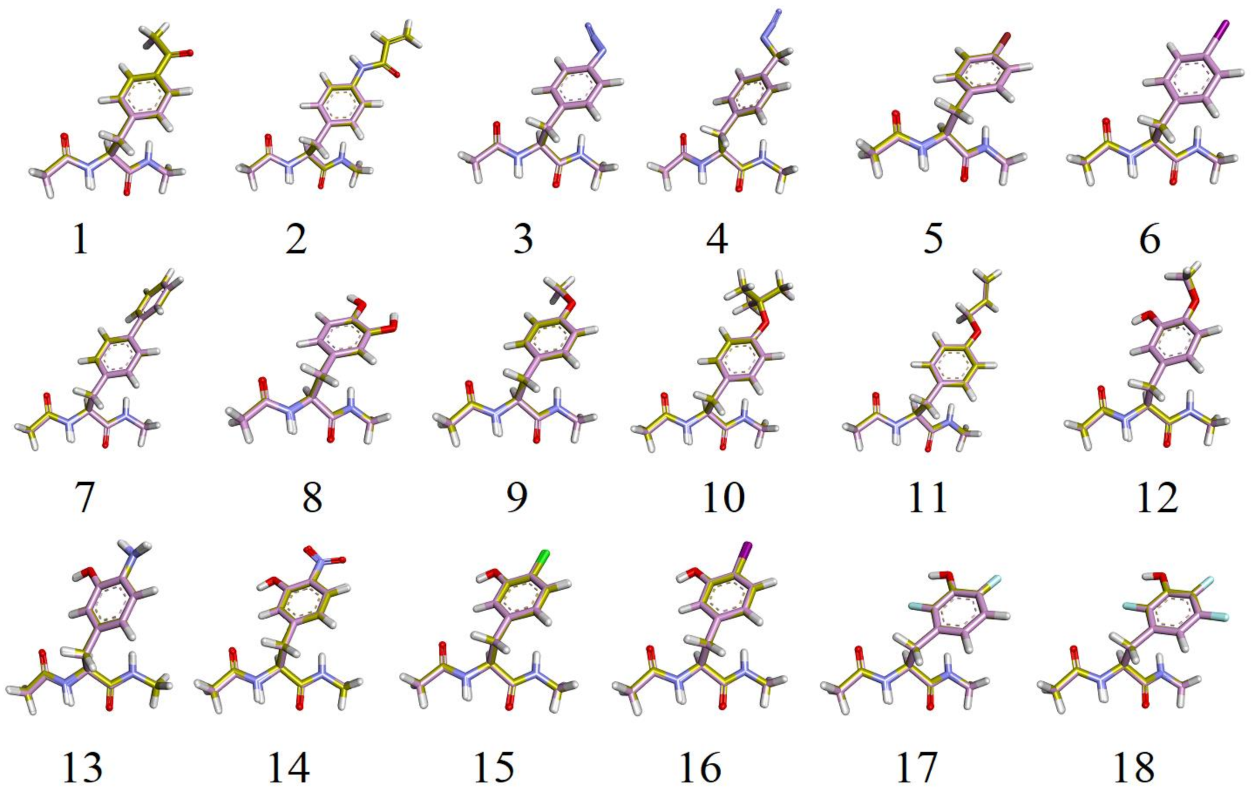

Figure S1. Overlap of 18 UAA β−backbone conformations after energy minimization of the QM(B3LYP/6−31G*) structures. N, O, and H atoms are shown in blue, red, and white, respectively. The C atoms of the simulation and QM structures are shown in pink and yellow, respectively. F(17 and 18), Cl(15), Br(5), and I(6 and 16) atoms are shown in cyan, green, red, and magenta, respectively.

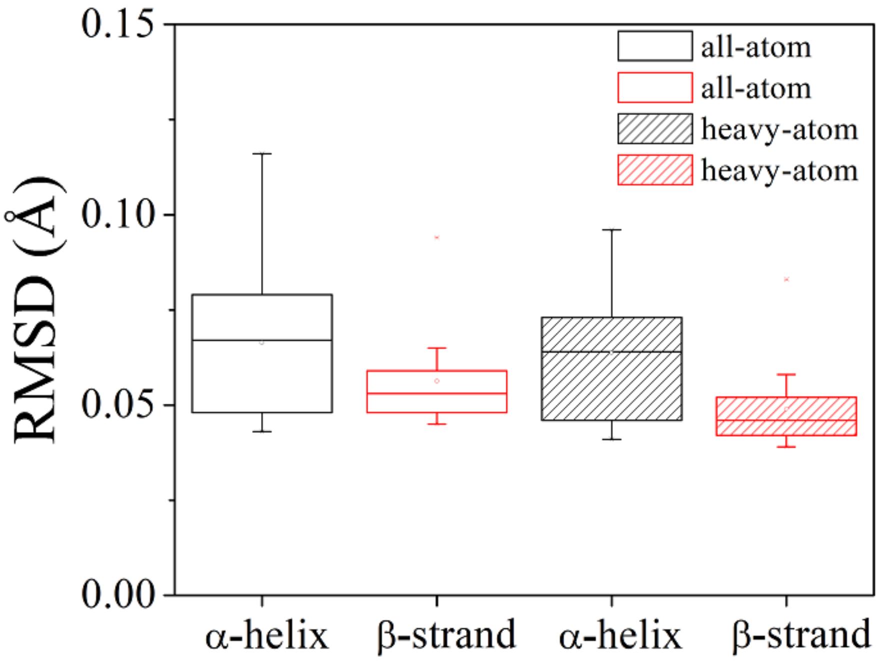

Figure S2. All−atom and heavy−atom RMSDs (Å) for the training set obtained from the initial parameters test.

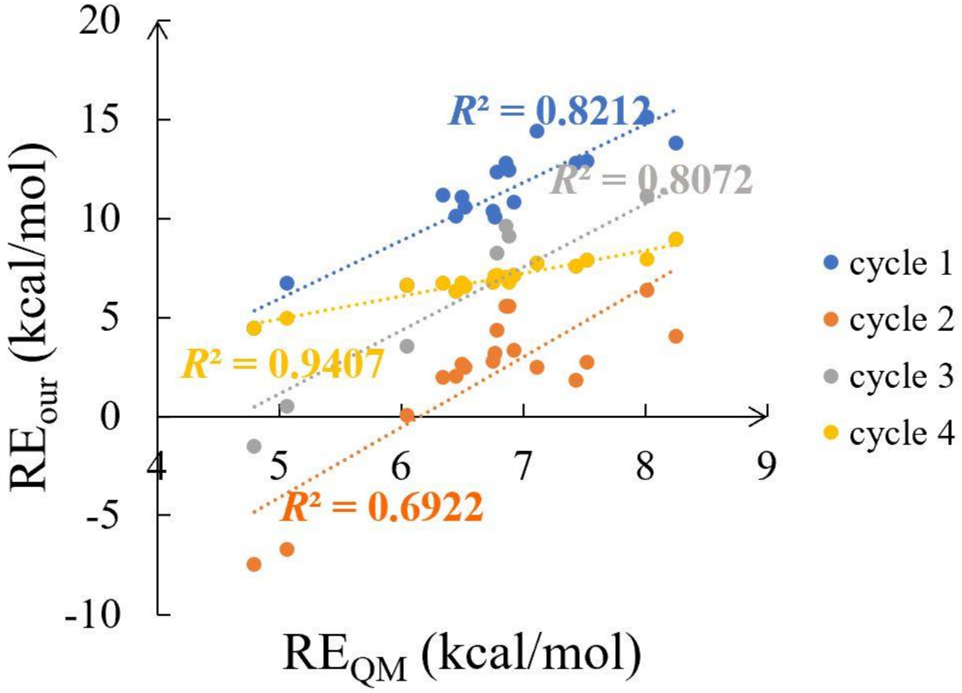

Figure S3. Correlation between the relative energies of 18 UAAs with α−/β−conformers obtained from QM calculations (MP2/cc−pVTZ) and our work. The blue, orange, gray, and yellow dots are taken from the cycle 1, 2, 3, and 4 parameters in our work, respectively.

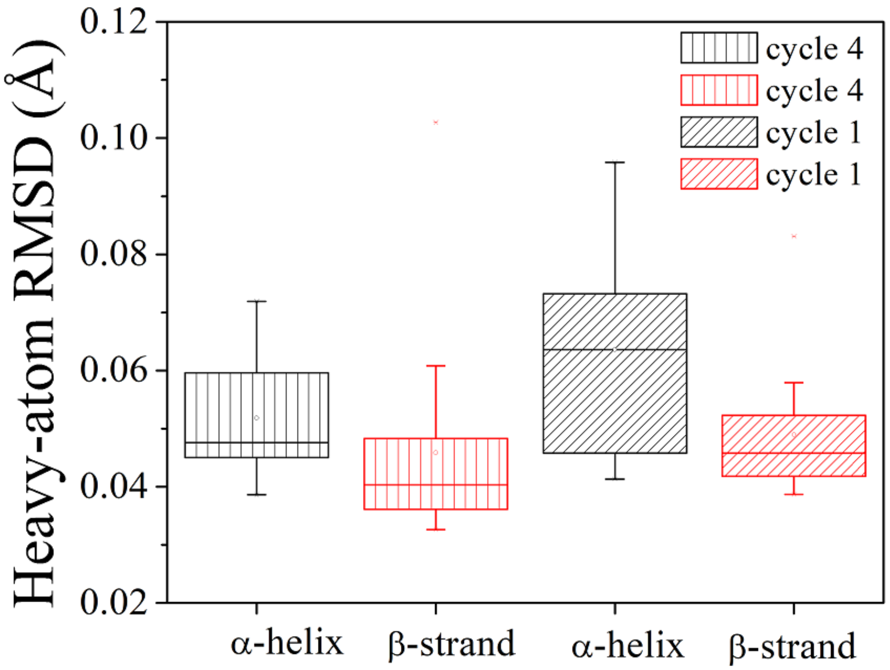

Figure S4. Heavy−atom RMSD distributions (Å) for 18 training sets analyzed after optimization from cycle−1 and cycle−4 (determined) parameters in our work.

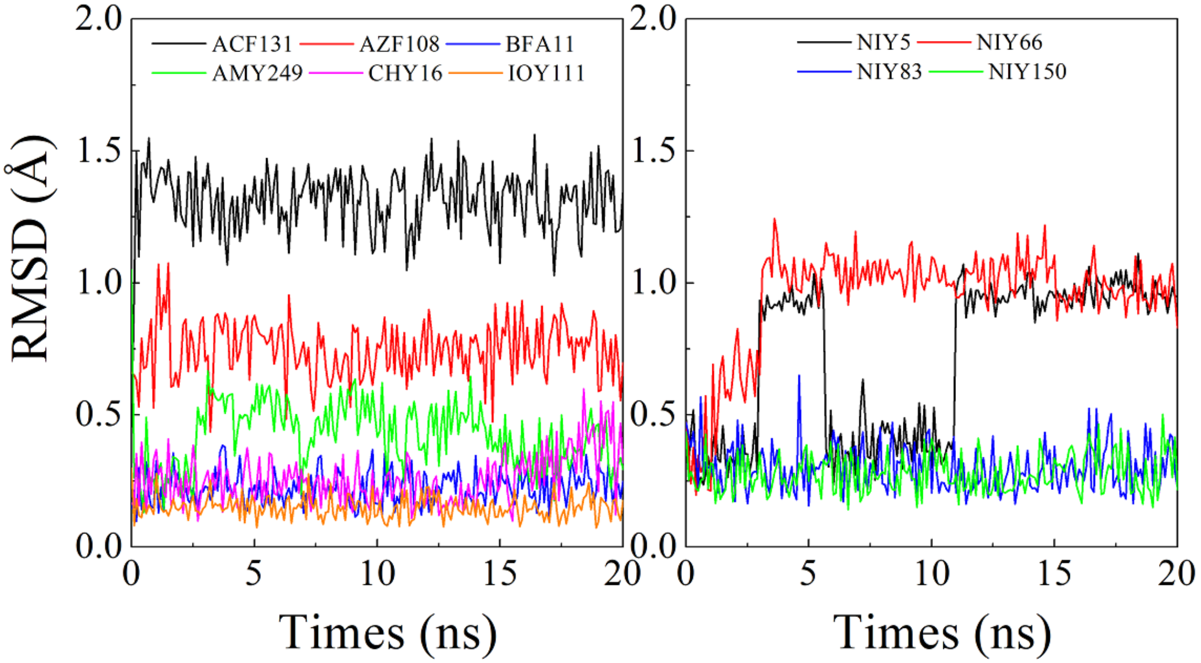

Figure S5. RMSD distributions as a function of time for UAAs incorporated into isolated proteins. For clarity, ACF131, AZF108, BFA11, AMY249, CHY16, and IOY111 are shown on the left, and NIY5, NIY66, NIY83, and NIY150 are depicted separately on the right.

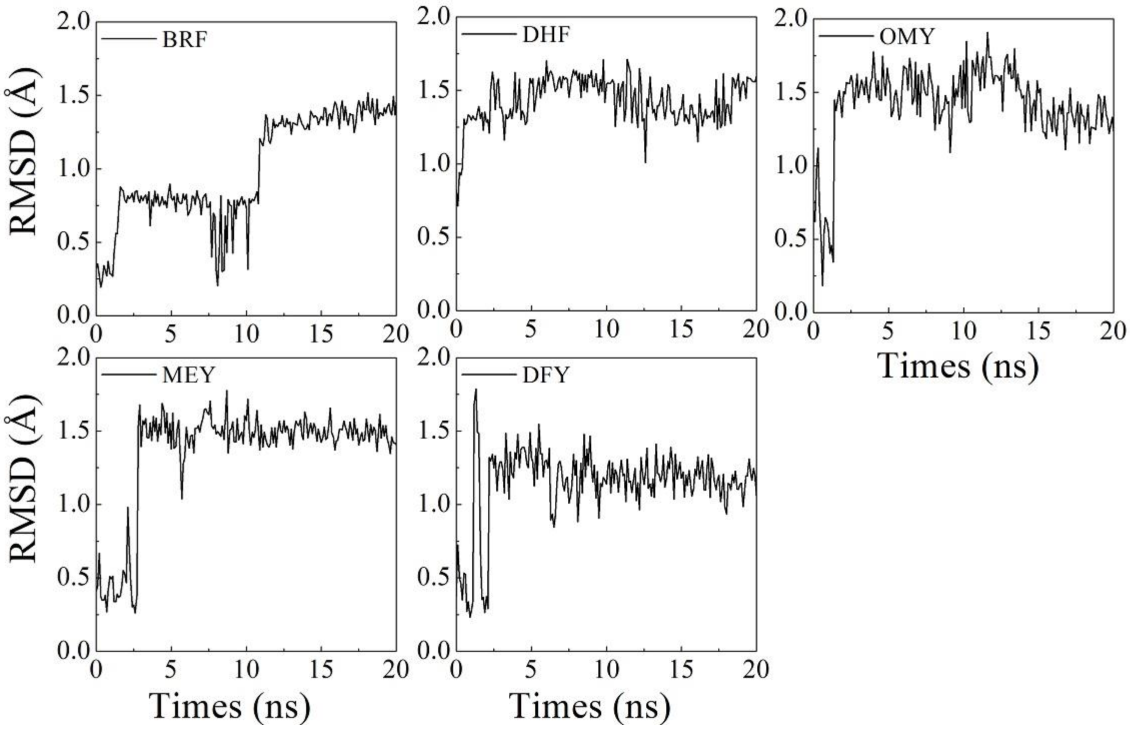

Figure S6. RMSD distributions over time for the UAAs BRF, DHF, OMY, MEY, and DFY as ligands.

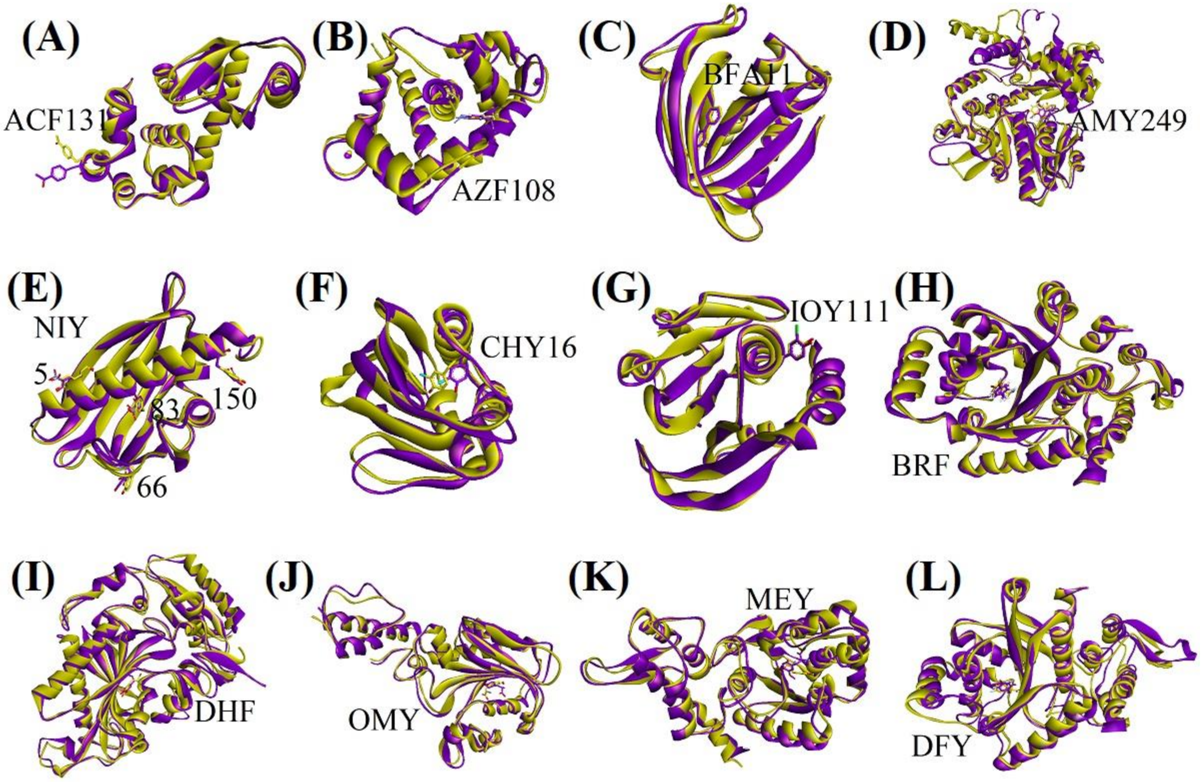

Figure S7. Stable whole structures after 20 ns MD simulations of (A) 3HWL, (B) 6HCS, (C) 4S0I, (D) 3MBB, (E) 4B9R, (F) 5D82, (J) 2Z10, (H) 2AG6, (I) 3TEG, (J) 3QTC, (K) 4HPW, and (L) 4HJX. The crystal and simulation structures are shown as yellow and purple representations, respectively. The C atoms of UAA are shown as sticks with the associated protein colors. The corresponding UAA names are labeled in black letters. The spheres in (B) represent Ca^{2+}.

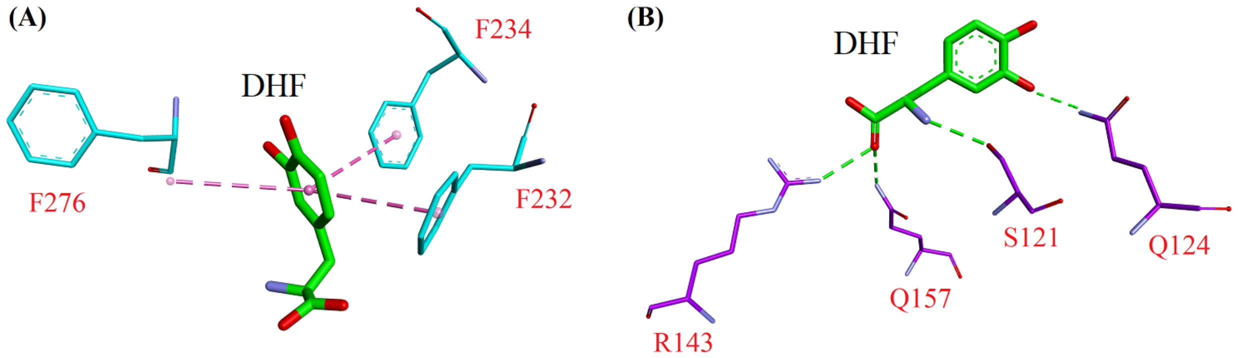

Figure S8. (A) Amide· · · π and π· · · π interactions between F232, F234, F276 and DHF predicted by MM/PBSA analysis; (B) Experimental H−bonded connections between S121, Q124, R143, and Q157 in the system of protein and DHF.

Supplementary Tables

Table S1. Collection of basic information for the 18 UAAs.[a]

No.	Name	Formula	Abbreviation here/reference	Position or role	PDB code
1	*p*−acetyl−phenylalanine	$C_{11}H_{13}NO_3$	ACF/*p*−AcPhe	131	3HWL
2	*p*−acrylamido−phenylalanine	$C_{12}H_{14}N_2O_3$	ARF/AcrF	216	4ZJ3
3	*p*−azido−phenylalanine	$C_9H_{10}N_4O_2$	AZF/AzF	108	6HCS
4	*p*−azidomethyl−phenylalanine	$C_{10}H_{12}N_4O_2$	AMF/AzMeF	−	−
5	*p*−bromo−l−phenylalanine	$C_9H_{10}BrNO_2$	BRF/*p*−BrPhe	ligand	2AG6
6	*p*−iodo−phenylalanine	$C_9H_{10}INO_2$	IOF/IF	4	4NTP
7	*p*−biphenylalanine	$C_{15}H_{15}NO_2$	BFA/BIF	11	4S0I
8	3,4−dihydroxy−l−phenylalanine	$C_9H_{11}NO_4$	DHF/L−dopa	ligand	3TEG
9	*o*−methyl−l−tyrosine	$C_{10}H_{13}NO_3$	OMY/OME	ligand	3QTC
10	*o*−tert−butyl−tyrosine	$C_{13}H_{19}NO_3$	OBY/OtBuY	−	−
11	*o*−allyl−tyrosine	$C_{12}H_{18}NO_3$	OAY/O−allyl−Y	−	−
12	3−methoxy−l−tyrosine	$C_{10}H_{13}NO_4$	MEY/3YM	ligand	4HPW
13	3−amino−l−tyrosine	$C_9H_{12}N_2O_3$	AMY/TY2	249	3MBB
14	3−nitro−l−tyrosine	$C_9H_{10}N_2O_5$	NIY/NIY	5, 66, 83, 150	4B9R
15	3−chloro−l−tyrosine	$C_9H_{10}ClNO_3$	CHY/Cl−Y	16	5D82
16	3−Iodo−l−tyrosine	$C_9H_{10}INO_3$	IOY/−	111	2Z10
17	3,5−difluoro−l−tyrosine	$C_9H_9F_2NO_3$	DFY/F2Y	ligand	4HJX
18	2,3,5−trifluoro tyrosine	$C_9H_8F_3NO_3$	TFY/−	−	−

[a] Charge parameters for systems 7, 9, 11, and 13 are also reported elsewhere(Khoury, Smadbeck et al. 2014). Systems 2 and 6 were removed due to the analog 4OV of serine (PDB ID: 4ZJ3) and cyclic peptide (PDB ID: 4NTP); thus, they were not included in this study.

Table S2. Relative energies (kcal/mol) of systems 7, 9, 11, and 13 obtained from MP2, the literature, and our work with absolute errors (δ).

System	RE_{MP2}	RE_{refer}	RE_{our}	δ_{refer}	δ_{our}
7	6.76	5.74	6.76	−1.02	0.00
9	6.86	8.52	7.00	1.66	0.14
11	6.79	8.05	7.10	1.26	0.31
13	8.26	4.40	8.94	−3.86	0.68
RMSD				2.25	0.38

Table S3. Terminal charge treatment for −H and −OH groups of BRF, DHF, OMY, MEY, and DFY as ligands.

Atom(s)	Charge				
	BRF	DHF	OMY	MEY	DFY
H	0.3385	0.3378	0.3367	0.3413	0.3426
OH	−0.6718	−0.6688	−0.6452	−0.6555	−0.6368
HO	0.3333	0.3310	0.3085	0.3142	0.2942

Table S4. The binding free energies (kcal/mol) of 2AG6 and their various components estimated using whole 20 ns, 12–20 ns, and 0–12 ns trajectories.

components	0–20ns	12–20ns	0–12ns
ΔE_{vdW}	–25.2(0.4)	–26.1(0.6)	–24.5(0.4)
ΔE_{ele}	–11.8(1.5)	–17.5(1.1)	–7.6(1.5)
$\Delta G_{pb/solv}$	33.6(1.5)	39.5(0.9)	29.2(1.6)
$\Delta G_{np/solv}$	–3.0(0.0)	–3.0(0.1)	–3.0(0.0)
ΔG_{pb}	21.8(0.8)	22.0(0.1)	21.6(0.1)
ΔG_{np}	–28.2(0.0)	–29.1(0.3)	–27.5(0.2)
ΔE_{MM}	–37.0(0.2)	–43.6(0.3)	–32.1(0.6)
ΔG_{solv}	30.6(0.8)	36.5(0.5)	26.2(0.9)
ΔG_{bind}	–6.4(1.0)	–7.1(1.0)	–5.9(1.1)

Modified Charge Parameters for 18 UAAs (in GROMACS format)

```
[ ACF ]
 [ atoms ]
    N    N        −0.592968    1
    H    H         0.328065    2
   CA   CX         0.045321    3
   HA   H1         0.089993    4
   CB   CT        −0.161169    5
  HB1   HC         0.080057    6
  HB2   HC         0.080057    7
   CG   CA         0.086801    8
  CD1   CA        −0.169579    9
  HD1   HA         0.145077   10
  CE1   CA        −0.152884   11
  HE1   HA         0.158781   12
   CZ   CA        −0.084043   13
   CH   C          0.665645   14
   OH   O         −0.548030   15
   CQ   CT        −0.424218   16
  HQ1   HC         0.114749   17
  HQ2   HC         0.114749   18
  HQ3   HC         0.114749   19
  CE2   CA        −0.152884   20
  HE2   HA         0.158781   21
  CD2   CA        −0.169579   22
  HD2   HA         0.145077   23
    C    C         0.687732   24
    O    O        −0.560279   25
 [ bonds ]
    N    H
    N   CA
   CA   HA
   CA   CB
   CA    C
   CB  HB1
   CB  HB2
   CB   CG
   CG  CD1
   CG  CD2
  CD1  HD1
  CD1  CE1
  CE1  HE1
  CE1   CZ
   CZ   CH
   CZ  CE2
```

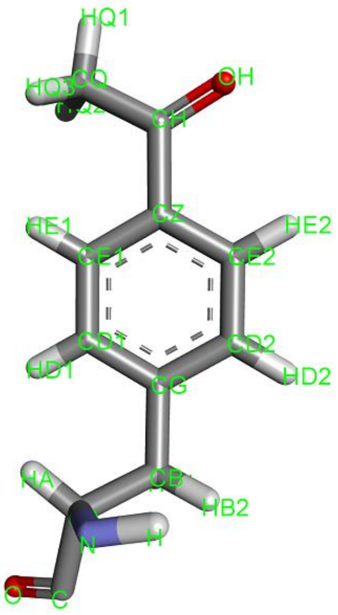

CH OH
CH CQ
CQ HQ1
CQ HQ2
CQ HQ3
CE2 HE2
CE2 CD2
CD2 HD2
C O
−C N

[impropers]

−C	CA	N	H
CA	+N	C	O
CG	CE2	CD2	HD2
CZ	CD2	CE2	HE2
CE1	CE2	CZ	CH
CZ	CQ	CH	OH
CD1	CZ	CE1	HE1
CG	CE1	CD1	HD1
CD1	CD2	CG	CB

[ARF]

[atoms]

N	N	−0.600072	1
H	H	0.326543	2
CA	CX	0.017227	3
HA	H1	0.089688	4
CB	CT	−0.108095	5
HB1	HC	0.062760	6
HB2	HC	0.062760	7
CG	CA	0.020568	8
CD1	CA	−0.146464	9
HD1	HA	0.155260	10
CE1	CA	−0.276844	11
HE1	HA	0.181990	12
CZ	CA	0.317740	13
NZ	N	−0.594733	14
HNZ	HN	0.290134	15
CH	C	0.868264	16
OH	O	−0.593185	17
CI	CT	−0.234130	18
HI	HC	0.111133	19
CK	CT	−0.414520	20
HK1	HC	0.205452	21
HK2	HC	0.205452	22
CE2	CA	−0.276844	23

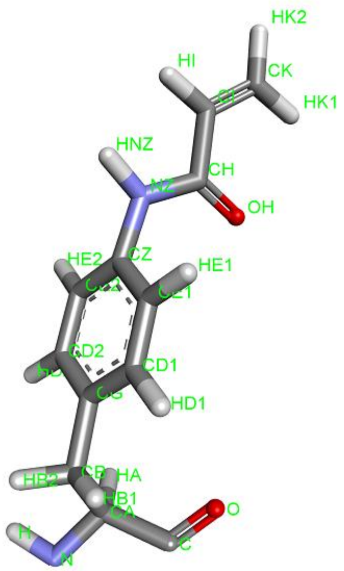

HE2 HA 0.181990 24
CD2 CA −0.146464 25
HD2 HA 0.155260 26
C C 0.709337 27
O O −0.570207 28

[bonds]

N H
N CA
CA HA
CA CB
CA C
CB HB1
CB HB2
CB CG
CG CD1
CG CD2
CD1 HD1
CD1 CE1
CE1 HE1
CE1 CZ
CZ NZ
CZ CE2
NZ HNZ
NZ CH
CH OH
CH CQ
CQ HQ1
CQ HQ2
CQ HQ3
CE2 HE2
CE2 CD2
CD2 HD2
C O
−C N

[impropers]

−C CA N H
CA +N C O
CG CE2 CD2 HD2
CZ CD2 CE2 HE2
CE1 CE2 CZ NZ
CZ CH NZ HNZ
CD1 CZ CE1 HE1
CG CE1 CD1 HD1
CD1 CD2 CG CB

[AZF]

[atoms]

N	N	−0.611678	1
H	H	0.331747	2
CA	CX	0.030469	3
HA	H1	0.093333	4
CB	CT	−0.091245	5
HB1	HC	0.061951	6
HB2	HC	0.061951	7
CG	CA	−0.015193	8
CD1	CA	−0.120596	9
HD1	HA	0.154457	10
CE1	CA	−0.296325	11
HE1	HA	0.180302	12
CZ	CA	0.385820	13
NZ	N2	−0.525003	14
NH	N1	0.592559	15
NQ	N1	−0.286380	16
CE2	CA	−0.296325	17
HE2	HA	0.180302	18
CD2	CA	−0.120596	19
HD2	HA	0.154457	20
C	C	0.698745	21
O	O	−0.562751	22

[bonds]

N	H
N	CA
CA	HA
CA	CB
CA	C
CB	HB1
CB	HB2
CB	CG
CG	CD1
CG	CD2
CD1	HD1
CD1	CE1
CE1	HE1
CE1	CZ
CZ	NZ
CZ	CE2
NZ	NH
NH	NQ
CE2	HE2
CE2	CD2
CD2	HD2
C	O

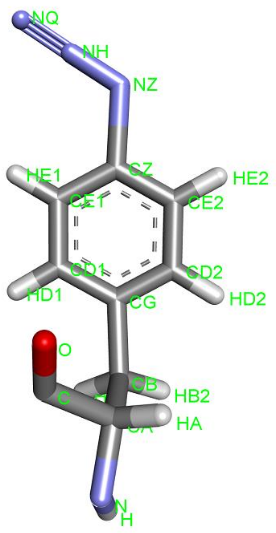

−C	N

[impropers]

−C	CA	N	H
CA	+N	C	O
CG	CE2	CD2	HD2
CZ	CD2	CE2	HE2
CE1	CE2	CZ	NZ
CD1	CZ	CE1	HE1
CG	CE1	CD1	HD1
CD1	CD2	CG	CB

[AMF]

[atoms]

N	N	−0.626935	1
H	H	0.335387	2
CA	CX	0.073311	3
HA	H1	0.099484	4
CB	CT	−0.094515	5
HB1	HC	0.058307	6
HB2	HC	0.058307	7
CG	CA	−0.011188	8
CD1	CA	−0.101535	9
HD1	HA	0.139019	10
CE1	CA	−0.245584	11
HE1	HA	0.156036	12
CZ	CA	0.108919	13
CH	CX	0.249550	14
HH1	H1	0.009399	15
HH2	H1	0.009399	16
NZ	N2	−0.586937	17
NH	N1	0.665834	18
NQ	N1	−0.341668	19
CE2	CA	−0.245584	20
HE2	HA	0.156036	21
CD2	CA	−0.101535	22
HD2	HA	0.139019	23
C	C	0.659982	24
O	O	−0.562506	25

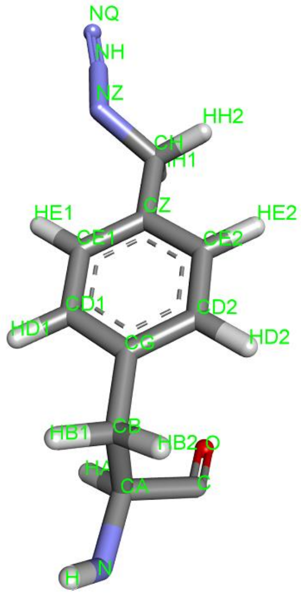

[bonds]

N	H
N	CA
CA	HA
CA	CB
CA	C
CB	HB1
CB	HB2

CB	CG
CG	CD1
CG	CD2
CD1	HD1
CD1	CE1
CE1	HE1
CE1	CZ
CZ	CH
CZ	CE2
CH	NZ
CH	HH1
CH	HH2
NZ	NH
NH	NQ
CE2	HE2
CE2	CD2
CD2	HD2
C	O
−C	N

[impropers]

−C	CA	N	H
CA	+N	C	O
CG	CE2	CD2	HD2
CZ	CD2	CE2	HE2
CE1	CE2	CZ	CH
CD1	CZ	CE1	HE1
CG	CE1	CD1	HD1
CD1	CD2	CG	CB

[BRF]

[atoms]

N	N	−0.636171	1
H	H	0.338465	2
CA	CX	0.132551	3
HA	H1	0.079717	4
CB	CT	−0.135354	5
HB1	HC	0.065186	6
HB2	HC	0.065186	7
CG	CA	0.078854	8
CD1	CA	−0.192723	9
HD1	HA	0.148556	10
CE1	CA	−0.024574	11
HE1	HA	0.128917	12
CZ	CA	−0.119466	13
BR	Br	−0.082556	14
CE2	CA	−0.024574	15

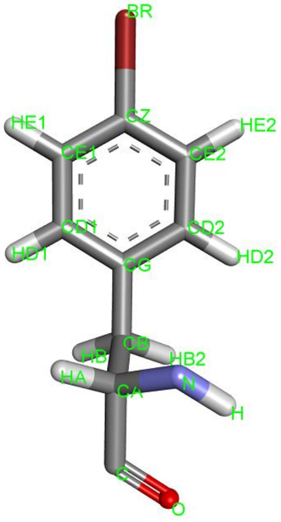

HE2 HA 0.128917 16
CD2 CA −0.192723 17
HD2 HA 0.148556 18
C C 0.642507 19
O O −0.549272 20
[bonds]
N H
N CA
CA HA
CA CB
CA C
CB HB1
CB HB2
CB CG
CG CD1
CG CD2
CD1 HD1
CD1 CE1
CE1 HE1
CE1 CZ
CZ BR
CZ CE2
CE2 HE2
CE2 CD2
CD2 HD2
C O
−C N
[impropers]
−C CA N H
CA +N C O
CG CE2 CD2 HD2
CZ CD2 CE2 HE2
CE1 CE2 CZ BR
CD1 CZ CE1 HE1
CG CE1 CD1 HD1
CD1 CD2 CG CB

[IOF]
[atoms]
N N −0.628224 1
H H 0.337194 2
CA CX 0.113409 3
HA H1 0.085752 4
CB CT −0.148074 5
HB1 HC 0.069706 6
HB2 HC 0.069706 7

CG CA 0.117705 8
CD1 CA −0.243530 9
HD1 HA 0.154680 10
CE1 CA 0.074550 11
HE1 HA 0.109479 12
CZ CA −0.252891 13
IZ I −0.054454 14
CE2 CA 0.074550 15
HE2 HA 0.109479 16
CD2 CA −0.243530 17
HD2 HA 0.154680 18
C C 0.651399 19
O O −0.551584 20

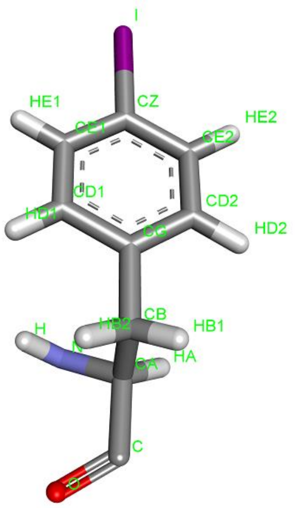

[bonds]
N H
N CA
CA HA
CA CB
CA C
CB HB1
CB HB2
CB CG
CG CD1
CG CD2
CD1 HD1
CD1 CE1
CE1 HE1
CE1 CZ
CZ IZ
CZ CE2
CE2 HE2
CE2 CD2
CD2 HD2
C O
−C N

[impropers]
−C CA N H
CA +N C O
CG CE2 CD2 HD2
CZ CD2 CE2 HE2
CE1 CE2 CZ IZ
CD1 CZ CE1 HE1
CG CE1 CD1 HD1
CD1 CD2 CG CB

[BFA]

[atoms]

N	N	−0.646125	1
H	H	0.337263	2
CA	CX	0.106099	3
HA	H1	0.081914	4
CB	CT	−0.075537	5
HB1	HC	0.050012	6
HB2	HC	0.050012	7
CG	CA	0.022083	8
CD1	CA	−0.165073	9
HD1	HA	0.146862	10
CE1	CA	−0.169141	11
HE1	HA	0.139507	12
CZ	CP	0.056581	13
CH	CP	0.069410	14
CQ1	CA	−0.153060	15
HQ1	HA	0.130871	16
CI1	CA	−0.152556	17
HI1	HA	0.142639	18
CK	CA	−0.134531	19
HK	HA	0.137201	20
CI2	CA	−0.152556	21
HI2	HA	0.142639	22
CQ2	CA	−0.153060	23
HQ2	HA	0.130871	24
CE2	CA	−0.169141	25
HE2	HA	0.139507	26
CD2	CA	−0.165073	27
HD2	HA	0.146862	28
C	C	0.665975	29
O	O	−0.560452	30

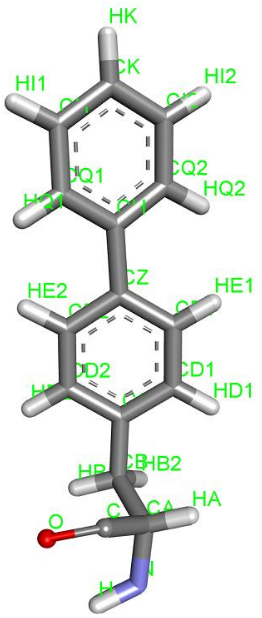

[bonds]

N	H
N	CA
CA	HA
CA	CB
CA	C
CB	HB1
CB	HB2
CB	CG
CG	CD1
CG	CD2
CD1	HD1
CD1	CE1
CE1	HE1
CE1	CZ

CZ CH
CZ CE2
CH CQ1
CH CQ2
CQ1 HQ1
CQ1 CI1
CI1 HI1
CI1 CK
CK HK
CK CI2
CI2 HI2
CI2 CQ2
CQ2 HQ2
CE2 HE2
CE2 CD2
CD2 HD2
C O
−C N

[impropers]

−C	CA	N	H
CA	+N	C	O
CG	CE2	CD2	HD2
CZ	CD2	CE2	HE2
CE1	CE2	CZ	CH
CD1	CZ	CE1	HE1
CG	CE1	CD1	HD1
CD1	CD2	CG	CB
CH	CI2	CQ2	HQ2
CK	CQ2	CI2	HI2
CI1	CI2	CK	HK
CQ1	CK	CI1	HI1
CH	CI1	CQ1	HQ1
CQ1	CQ2	CH	CZ

[DHF]

[atoms]

N	N	−0.631110	1
H	H	0.337773	2
CA	CX	−0.015382	3
HA	H1	0.110199	4
CB	CT	−0.033092	5
HB1	HC	0.055760	6
HB2	HC	0.055760	7
CG	CA	−0.037822	8
CD1	CA	−0.206742	9
HD1	HA	0.196805	10

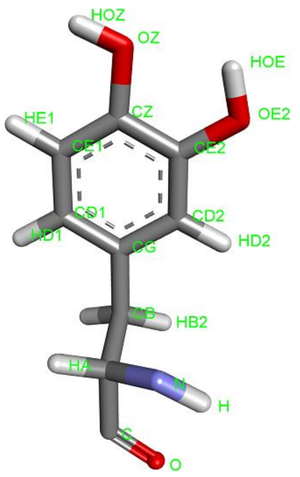

```
  CE1    CA         −0.321886   11
  HE1    HA          0.185188   12
   CZ    CA          0.240171   13
   OZ    OH         −0.663307   14
  HOZ    HO          0.471746   15
  CE2    CA          0.338255   16
  OE2    OH         −0.630550   17
  HOE    HO          0.473102   18
  CD2    CA         −0.265864   19
  HD2    HA          0.195348   20
    C    C           0.713620   21
    O    O          −0.567971   22
[ bonds ]
    N     H
    N    CA
   CA    HA
   CA    CB
   CA     C
   CB   HB1
   CB   HB2
   CB    CG
   CG   CD1
   CG   CD2
  CD1   HD1
  CD1   CE1
  CE1   HE1
  CE1    CZ
   CZ    OZ
   CZ   CE2
   OZ   HOZ
  CE2   OE2
  CE2   CD2
  OE2   HOE
  CD2   HD2
    C     O
   −C     N
[ impropers ]
   −C    CA     N     H
   CA    +N     C     O
   CG   CE2   CD2   HD2
   CZ   CD2   CE2   OE2
  CE1   CE2    CZ    OZ
  CD1    CZ   CE1   HE1
   CG   CE1   CD1   HD1
  CD1   CD2    CG    CB
```

[OMY]

[atoms]

N	N	−0.622198	1
H	H	0.336747	2
CA	CX	−0.000130	3
HA	H1	0.109103	4
CB	CT	−0.014055	5
HB1	HC	0.037469	6
HB2	HC	0.037469	7
CG	CA	−0.104189	8
CD1	CA	−0.073593	9
HD1	HA	0.140576	10
CE1	CA	−0.357275	11
HE1	HA	0.177307	12
CZ	CA	0.485419	13
OH	OS	−0.388852	14
CQ	CX	0.012382	15
HQ1	H1	0.064859	16
HQ2	H1	0.064859	17
HQ3	H1	0.064859	18
CE2	CA	−0.357275	19
HE2	HA	0.177307	20
CD2	CA	−0.073593	21
HD2	HA	0.140576	22
C	C	0.707350	23
O	O	−0.565125	24

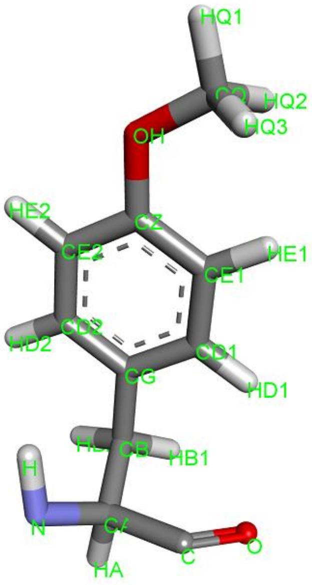

[bonds]

N	H
N	CA
CA	HA
CA	CB
CA	C
CB	HB1
CB	HB2
CB	CG
CG	CD1
CG	CD2
CD1	HD1
CD1	CE1
CE1	HE1
CE1	CZ
CZ	OH
CZ	CE2
OH	CQ
CQ	HQ1
CQ	HQ2

CQ HQ3
CE2 HE2
CE2 CD2
CD2 HD2
C O
−C N

[impropers]

−C	CA	N	H
CA	+N	C	O
CG	CE2	CD2	HD2
CZ	CD2	CE2	HE2
CE1	CE2	CZ	OH
CD1	CZ	CE1	HE1
CG	CE1	CD1	HD1
CD1	CD2	CG	CB

[OBY]

[atoms]

N	N	−0.610229	1
H	H	0.333212	2
CA	CX	−0.005394	3
HA	H1	0.112488	4
CB	CT	−0.034506	5
HB1	HC	0.040622	6
HB2	HC	0.040622	7
CG	CA	−0.054502	8
CD1	CA	−0.107156	9
HD1	HA	0.141610	10
CE1	CA	−0.339408	11
HE1	HA	0.189885	12
CZ	CA	0.459208	13
OH	OS	−0.529831	14
CQ	CT	0.730425	15
CI1	CT	−0.407582	16
HI11	HC	0.092757	17
HI12	HC	0.092757	18
HI13	HC	0.092757	19
CI2	CT	−0.407582	20
HI21	HC	0.092757	21
HI22	HC	0.092757	22
HI23	HC	0.092757	23
CI3	CT	−0.407582	24
HI31	HC	0.092757	25
HI32	HC	0.092757	26
HI33	HC	0.092757	27
CE2	CA	−0.339408	28

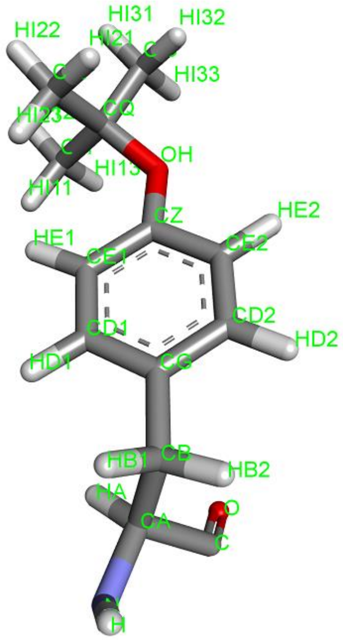

HE2	HA	0.189885	29
CD2	CA	−0.107156	30
HD2	HA	0.141610	31
C	C	0.702909	32
O	O	−0.566958	33

[bonds]

N	H
N	CA
CA	HA
CA	CB
CA	C
CB	HB1
CB	HB2
CB	CG
CG	CD1
CG	CD2
CD1	HD1
CD1	CE1
CE1	HE1
CE1	CZ
CZ	OH
CZ	CE2
OH	CQ
CQ	CI1
CQ	CI2
CQ	CI3
CI1	HI11
CI1	HI12
CI1	HI13
CI2	HI21
CI2	HI22
CI2	HI23
CI3	HI31
CI3	HI32
CI3	HI33
CE2	HE2
CE2	CD2
CD2	HD2
C	O
−C	N

[impropers]

−C	CA	N	H
CA	+N	C	O
CG	CE2	CD2	HD2
CZ	CD2	CE2	HE2
CE1	CE2	CZ	OH

CD1	CZ	CE1	HE1
CG	CE1	CD1	HD1
CD1	CD2	CG	CB

[OAY]

[atoms]

N	N	−0.617181	1
H	H	0.331605	2
CA	CX	0.004668	3
HA	H1	0.100334	4
CB	CT	−0.033346	5
HB1	HC	0.044549	6
HB2	HC	0.044549	7
CG	CA	−0.082531	8
CD1	CA	−0.083691	9
HD1	HA	0.144979	10
CE1	CA	−0.342035	11
HE1	HA	0.171306	12
CZ	CA	0.438428	13
OH	OS	−0.403116	14
CQ	CX	0.253577	15
HQ1	H1	0.023603	16
HQ2	H1	0.023603	17
CI	C2	−0.134131	18
HI1	HA	0.146957	19
CK	C2	−0.459426	20
HK2	HA	0.196243	21
HK3	HA	0.196243	22
CE2	CA	−0.342035	23
HE2	HA	0.171306	24
CD2	CA	−0.083691	25
HD2	HA	0.144979	26
C	C	0.714081	27
O	O	−0.569827	28

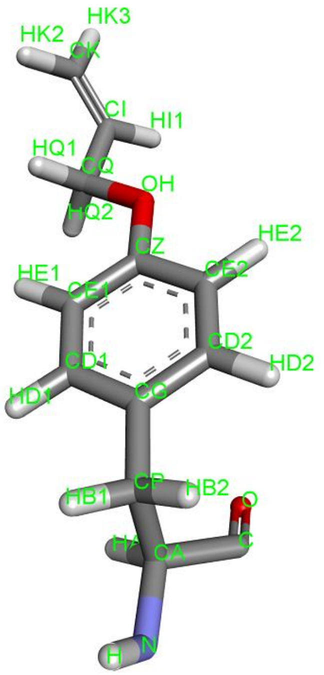

[bonds]

N	H
N	CA
CA	HA
CA	CB
CA	C
CB	HB1
CB	HB2
CB	CG
CG	CD1
CG	CD2
CD1	HD1

CD1	CE1
CE1	HE1
CE1	CZ
CZ	OH
CZ	CE2
OH	CQ
CQ	HQ2
CQ	HQ3
CQ	CI
CI	HI1
CI	CK
CK	HK2
CK	HK3
CE2	HE2
CE2	CD2
CD2	HD2
C	O
−C	N

[impropers]

−C	CA	N	H
CA	+N	C	O
CG	CE2	CD2	HD2
CZ	CD2	CE2	HE2
CE1	CE2	CZ	OH
CD1	CZ	CE1	HE1
CG	CE1	CD1	HD1
CD1	CD2	CG	CB
CQ	CK	CI	HI1

[MEY]

[atoms]

N	N	−0.637492	1
H	H	0.341322	2
CA	CX	−0.000362	3
HA	H1	0.123571	4
CB	CT	−0.007303	5
HB1	HC	0.036440	6
HB2	HC	0.036440	7
CG	CA	0.034691	8
CD1	CA	−0.161411	9
HD1	HA	0.138689	10
CE1	CA	−0.239675	11
HE1	HA	0.175509	12
CZ	CA	0.245599	13
OZ	OH	−0.573584	14
HOZ	HO	0.428338	15

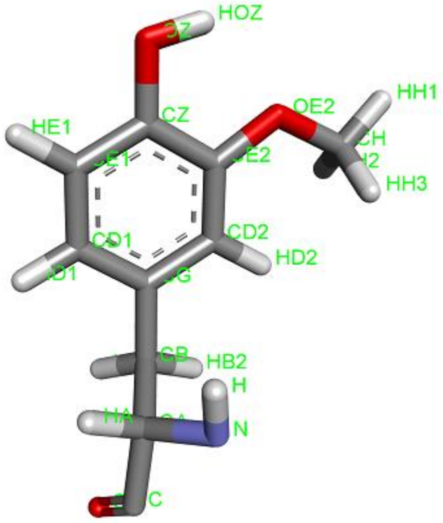

CE2	CA	0.332708	16
OE2	OS	−0.378273	17
CH	CX	0.044247	18
HH1	H1	0.057392	19
HH2	H1	0.057392	20
HH3	H1	0.057392	21
CD2	CA	−0.462456	22
HD2	HA	0.212064	23
C	C	0.700916	24
O	O	−0.562153	25

[bonds]

N	H
N	CA
CA	HA
CA	CB
CA	C
CB	HB1
CB	HB2
CB	CG
CG	CD1
CG	CD2
CD1	HD1
CD1	CE1
CE1	HE1
CE1	CZ
CZ	OZ
CZ	CE2
OZ	CH
CH	HH1
CH	HH2
CH	HH3
CE2	OE2
CE2	CD2
OE2	HOE
CD2	HD2
C	O
−C	N

[impropers]

−C	CA	N	H
CA	+N	C	O
CG	CE2	CD2	HD2
CZ	CD2	CE2	OE2
CE1	CE2	CZ	OZ
CD1	CZ	CE1	HE1
CG	CE1	CD1	HD1
CD1	CD2	CG	CB

[AMY]

[atoms]

N	N	−0.679543	1
H	H	0.347235	2
CA	CX	0.064636	3
HA	H1	0.093048	4
CB	CT	−0.047949	5
HB1	HC	0.049128	6
HB2	HC	0.049128	7
CG	CA	0.028915	8
CD1	CA	−0.159842	9
HD1	HA	0.143381	10
CE1	CA	−0.347005	11
HE1	HA	0.197913	12
CZ	CA	0.323461	13
OZ	OH	−0.604046	14
HOZ	HO	0.433916	15
CE2	CA	0.331894	16
NE2	NH	−0.885069	17
HN1	HN	0.377089	18
HN2	HN	0.377089	19
CD2	CA	−0.475834	20
HD2	HA	0.239784	21
C	C	0.708023	22
O	O	−0.565348	23

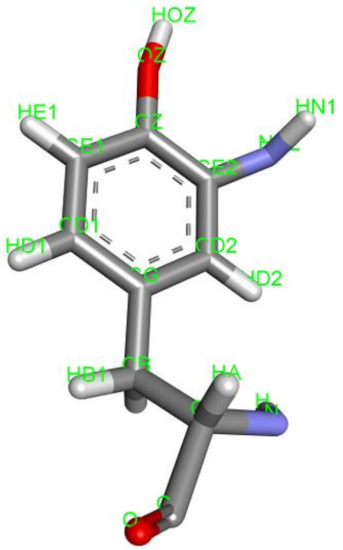

[bonds]

N	H
N	CA
CA	HA
CA	CB
CA	C
CB	HB1
CB	HB2
CB	CG
CG	CD1
CG	CD2
CD1	HD1
CD1	CE1
CE1	HE1
CE1	CZ
CZ	OZ
CZ	CE2
OZ	HOZ
CE2	NE2
CE2	CD2

NE2 HN1
NE2 HN2
CD2 HD2
C O
−C N

[impropers]

−C CA N H
CA +N C O
CG CE2 CD2 HD2
CZ CD2 CE2 NE2
CE1 CE2 CZ OZ
CD1 CZ CE1 HE1
CG CE1 CD1 HD1
CD1 CD2 CG CB

[NIY]

[atoms]

N	N	−0.628855	1
H	H	0.344263	2
CA	CX	0.057639	3
HA	H1	0.102463	4
CB	CT	−0.140789	5
HB1	HC	0.082099	6
HB2	HC	0.082099	7
CG	CA	0.115874	8
CD1	CA	−0.108660	9
HD1	HA	0.127077	10
CE1	CA	−0.239150	11
HE1	HA	0.201096	12
CZ	CA	−0.016727	13
OZ	OH	−0.514320	14
HOZ	HO	0.419576	15
CE2	CA	0.376401	16
NE	NO	0.729319	17
OE1	O	−0.445371	18
OE2	O	−0.445371	19
CD2	CA	−0.453573	20
HD2	HA	0.229708	21
C	C	0.677638	22
O	O	−0.552436	23

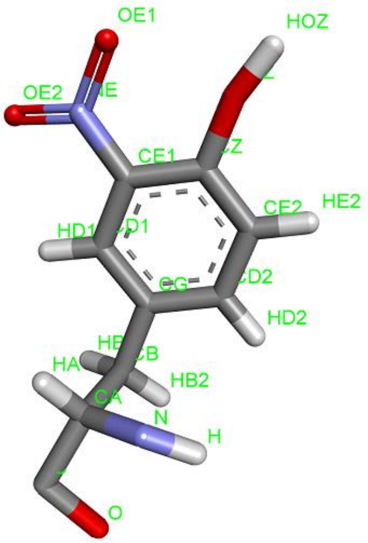

[bonds]

N H
N CA
CA HA
CA CB
CA C

CB HB1
CB HB2
CB CG
CG CD1
CG CD2
CD1 HD1
CD1 CE1
CE1 HE1
CE1 CZ
CZ OZ
CZ CE2
OZ HOZ
CE2 NE
CE2 CD2
NE OE1
NE OE2
CD2 HD2
C O
−C N

[impropers]

−C	CA	N	H
CA	+N	C	O
CG	CE2	CD2	HD2
CZ	CD2	CE2	NE
OE1	OE2	NE	CE2
CE1	CE2	CZ	OZ
CD1	CZ	CE1	HE1
CG	CE1	CD1	HD1
CD1	CD2	CG	CB

[CHY]

[atoms]

N	N	−0.633506	1
H	H	0.340552	2
CA	CX	0.061315	3
HA	H1	0.100284	4
CB	CT	−0.148201	5
HB1	HC	0.078177	6
HB2	HC	0.078177	7
CG	CA	0.167853	8
CD1	CA	−0.233046	9
HD1	HA	0.144833	10
CE1	CA	−0.066292	11
HE1	HA	0.148944	12
CZ	CA	−0.142250	13
OZ	OH	−0.643888	14

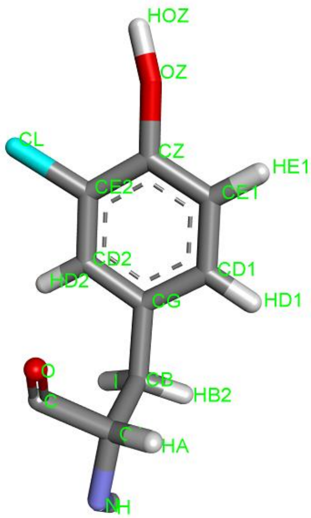

```
  HOZ    HO        0.454001   15
  CE2    CA        0.577262   16
   CL    Cl       −0.082535   17
  CD2    CA       −0.555127   18
  HD2    HA        0.231728   19
    C    C         0.679093   20
    O    O        −0.557374   21
 [ bonds ]
    N     H
    N    CA
   CA    HA
   CA    CB
   CA     C
   CB   HB1
   CB   HB2
   CB    CG
   CG   CD1
   CG   CD2
  CD1   HD1
  CD1   CE1
  CE1   HE1
  CE1    CZ
   CZ    OZ
   CZ   CE2
   OZ   HOZ
  CE2    CL
  CE2   CD2
  CD2   HD2
    C     O
   −C     N
 [ impropers ]
   −C    CA     N     H
   CA    +N     C     O
   CG   CE2   CD2   HD2
   CZ   CD2   CE2    CL
  CE1   CE2    CZ    OZ
  CD1    CZ   CE1   HE1
   CG   CE1   CD1   HD1
  CD1   CD2    CG    CB

[ IOY ]
 [ atoms ]
    N    N        −0.628817    1
    H    H         0.340025    2
   CA    CX        0.080468    3
   HA    H1        0.098039    4
```

CB CT −0.191222 5
HB1 HC 0.087008 6
HB2 HC 0.087008 7
CG CA 0.242398 8
CD1 CA −0.600876 9
HD1 HA 0.223155 10
CE1 CA 0.724183 11
IE I −0.006281 12
CZ CA −0.409140 13
OZ OH −0.685158 14
HOZ HO 0.467139 15
CE2 CA 0.103927 16
HE2 HA 0.116305 17
CD2 CA −0.310852 18
HD2 HA 0.150317 19
C C 0.665391 20
O O −0.553014 21

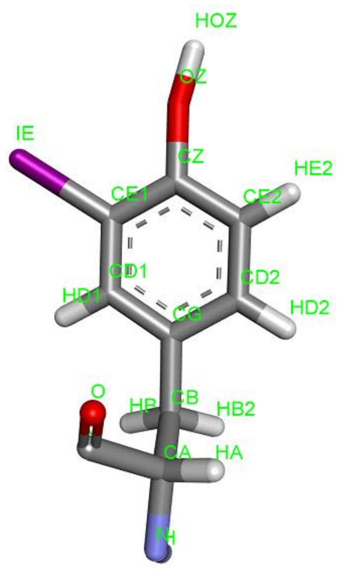

[bonds]
N H
N CA
CA HA
CA CB
CA C
CB HB1
CB HB2
CB CG
CG CD1
CG CD2
CD1 HD1
CD1 CE1
CE1 IE
CE1 CZ
CZ OZ
CZ CE2
OZ HOZ
CE2 HE2
CE2 CD2
CD2 HD2
C O
−C N
[impropers]
−C CA N H
CA +N C O
CG CE2 CD2 HD2
CZ CD2 CE2 HE2
CE1 CE2 CZ OZ

CD1	CZ	CE1	IE
CG	CE1	CD1	HD1
CD1	CD2	CG	CB

[DFY]

[atoms]

N	N	−0.624857	1
H	H	0.342603	2
CA	CX	0.031501	3
HA	H1	0.101052	4
CB	CT	−0.062253	5
HB1	HC	0.081422	6
HB2	HC	0.081422	7
CG	CA	−0.077706	8
CD1	CA	−0.170488	9
HD1	HA	0.188549	10
CE1	CA	0.288912	11
FE1	F	−0.179252	12
CZ	CA	0.186830	13
OZ	OH	−0.553477	14
HOZ	HO	0.435800	15
CE2	CA	0.103588	16
FE2	F	−0.155011	17
CD2	CA	−0.347925	18
HD2	HA	0.214698	19
C	C	0.670294	20
O	O	−0.555702	21

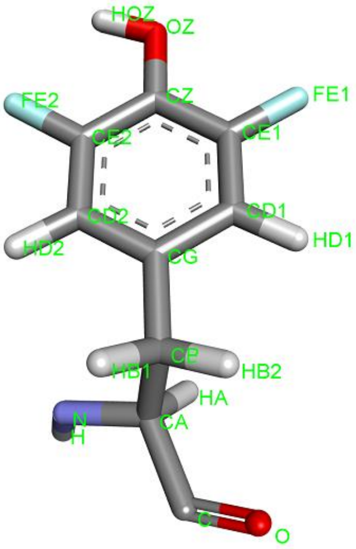

[bonds]

N	H
N	CA
CA	HA
CA	CB
CA	C
CB	HB1
CB	HB2
CB	CG
CG	CD1
CG	CD2
CD1	HD1
CD1	CE1
CE1	FE1
CE1	CZ
CZ	OZ
CZ	CE2
OZ	HOZ
CE2	FE2

CE2 CD2
CD2 HD2
C O
−C N

[impropers]

−C CA N H
CA +N C O
CG CE2 CD2 HD2
CZ CD2 CE2 FE2
CE1 CE2 CZ OZ
CD1 CZ CE1 FE1
CG CE1 CD1 HD1
CD1 CD2 CG CB

[TFY]

[atoms]

N	N	−0.636885	1
H	H	0.347539	2
CA	CX	−0.045299	3
HA	H1	0.137231	4
CB	CT	0.046971	5
HB1	HC	0.058086	6
HB2	HC	0.058086	7
CG	CA	−0.101327	8
CD1	CA	−0.322669	9
HD1	HA	0.224769	10
CE1	CA	0.229299	11
FE1	F	−0.175634	12
CZ	CA	0.224831	13
OZ	OH	−0.542554	14
HOZ	HO	0.439426	15
CE2	CA	0.136910	16
FE2	F	−0.141515	17
CD2	CA	0.066534	18
FD2	F	−0.156758	19
C	C	0.714423	20
O	O	−0.561463	21

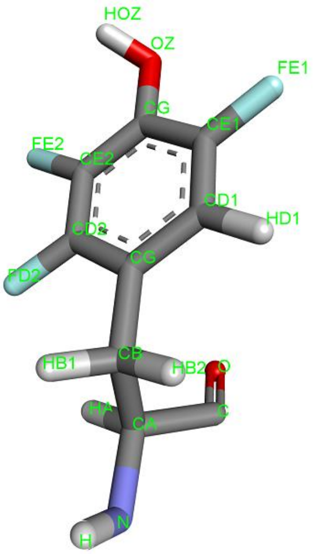

[bonds]

N H
N CA
CA HA
CA CB
CA C
CB HB1
CB HB2
CB CG

CG CD1
CG CD2
CD1 HD1
CD1 CE1
CE1 FE1
CE1 CZ
CZ OZ
CZ CE2
OZ HOZ
CE2 FE2
CE2 CD2
CD2 FD2
C O
−C N
[impropers]
−C CA N H
CA +N C O
CG CE2 CD2 FD2
CZ CD2 CE2 FE2
CE1 CE2 CZ OZ
CD1 CZ CE1 FE1
CG CE1 CD1 HD1
CD1 CD2 CG CB
